# A Live-Attenuated HSV-2 *ICP0* ^−^ Virus Elicits 10 to 100 Times Greater Protection against Genital Herpes than a Glycoprotein D Subunit Vaccine

**DOI:** 10.1371/journal.pone.0017748

**Published:** 2011-03-11

**Authors:** William P. Halford, Ringo Püschel, Edward Gershburg, Andrew Wilber, Svetlana Gershburg, Brandon Rakowski

**Affiliations:** Department of Microbiology and Immunology, Southern Illinois University School of Medicine, Springfield, Illinois, United States of America; University of Nebraska Medical Center, United States of America

## Abstract

Glycoprotein D (gD-2) is the entry receptor of herpes simplex virus 2 (HSV-2), and is the immunogen in the pharmaceutical industry's lead HSV-2 vaccine candidate. Efforts to prevent genital herpes using gD-2 subunit vaccines have been ongoing for 20 years at a cost in excess of $100 million. To date, gD-2 vaccines have yielded equivocal protection in clinical trials. Therefore, using a small animal model, we sought to determine if a live-attenuated HSV-2 *ICP0* ^−^ virus would elicit better protection against genital herpes than a gD-2 subunit vaccine. Mice immunized with gD-2 and a potent adjuvant (alum+monophosphoryl lipid A) produced high titers of gD-2 antibody. While gD-2-immunized mice possessed significant resistance to HSV-2, only 3 of 45 gD-2-immunized mice survived an overwhelming challenge of the vagina or eyes with wild-type HSV-2 (MS strain). In contrast, 114 of 115 mice immunized with a live HSV-2 *ICP0* ^−^ virus, 0ΔNLS, survived the same HSV-2 MS challenges. Likewise, 0ΔNLS-immunized mice shed an average 125-fold less HSV-2 MS challenge virus per vagina relative to gD-2-immunized mice. *In vivo* imaging demonstrated that a luciferase-expressing HSV-2 challenge virus failed to establish a detectable infection in 0ΔNLS-immunized mice, whereas the same virus readily infected naïve and gD-2-immunized mice. Collectively, these results suggest that a HSV-2 vaccine might be more likely to prevent genital herpes if it contained a live-attenuated HSV-2 virus rather than a single HSV-2 protein.

## Introduction

Infections with herpes simplex virus 2 (HSV-2) are exceedingly common; ∼1 billion people serve as carriers of HSV-2 and ∼20 million people acquire new HSV-2 infections each year [1]. An effective HSV-2 vaccine would be useful in breaking the cycle, and protecting young adults from the 1 in 10 chance that they will acquire HSV-2 before they marry [2], [3], [4].

Efforts to develop an HSV-2 vaccine have long been predicated on the assumption that a live-attenuated HSV-2 virus would be too dangerous to use as a human vaccine. Thus, four types of vaccines have been most carefully considered: ***1.*** HSV-2 subunit vaccines [5], [6], [7], [8], [9], [10]; ***2.*** gene-delivery vehicles that express HSV-2 proteins [11], [12], [13], [14], [15], [16], [17], [18], [19]; ***3.*** replication-defective HSV-2 viruses [20], [21], [22], [23], [24], [25]; and ***4.*** HSV-2 viruses that are overattenuated and/or unable to replicate in neurons [26], [27], [28], [29], [30], [31].

Of these approaches, subunit vaccines based on a combination of HSV-2 glycoprotein D (gD-2) and a potent adjuvant have received the greatest level of consideration. The technology to produce recombinant gD-2 emerged in the early 1980s [32], and the formulation of gD-2 subunit vaccines has undergone a continual process of testing and refinement ever since [6], [7], [11], [19], [33], [34], [35], [36], [37], [38], [39], [40], [41]. Mouse and guinea pig studies have, and continue, to serve as a testing ground for identifying the optimum combination of gD-2 immunogen and adjuvant [6], [37], [38], [42]. Two clinical trials of gD-2 subunit vaccines were completed in the late 1990s and early 2000s [36], [41]. In the latter trial, it was noted that HSV-1 seronegative women responded to a gD-2 subunit vaccine with significant reductions in the rate of acquiring HSV-2 genital herpes [36]. These results offered hope that vaccine-induced protection against HSV-2 genital herpes was possible, but would need to be further improved.

Mature gD-2 is a 368-amino-acid protein with a hydrophobic C-terminus. In nature, the gD-2 protein is embedded in the envelope of HSV-2 virions, and initiates viral entry by attaching to cell-surface receptors [43]. The recombinant gD-2 protein used in vaccines lacks the C-terminus, which allows the truncated protein to be secreted by producer cells [34], [35]. Hence, a 302-amino-acid gD-2 peptide (gD-2_302t_) is secreted from plasmid-transfected chinese hamster ovary cells [35], and it has served as the immunogen in human gD-2 subunit vaccines [36], [37], [38]. Likewise, a 306-amino-acid gD-2 peptide (gD-2_306t_) is secreted from baculovirus-infected insect cells [34] and exhibits similar immunogenic properties to gD-2_302t_ in animal studies [6]. Thus, the protein antigen used in gD-2 subunit vaccines has remained relatively constant over time.

The second critical component of a subunit vaccine is the adjuvant, which has repeatedly changed in gD-2 subunit vaccine formulations over the years. In its most recent formulation [6], [37], [38], gD-2 subunit vaccines relied on Glaxo Smith Kline's “adjuvant system 4” (AS04) [44], which means that gD-2 was absorbed to alum adjuvant and combined with monophosphoryl lipid A (MPL). MPL is derived from *Salmonella minnesota*, and contains a non-toxic analogue of lipid A (a toll-like receptor 4 agonist), which potently activates professional antigen-presenting cells [45], [46]. Hence, MPL drives more potent immune responses to protein antigens, and was instrumental in the success of the Gardasil® vaccine [47].

Given the acute need for a genital herpes vaccine, the National Institutes of Allergy and Infectious Disease (NIAID) invested $27.6 million into a gD-2 clinical trial that was conducted from 2003 to 2009 at 48 test sites in the United States [48]. The goal was to determine if the latest gD-2 subunit vaccine, Glaxo Smith Kline's Herpevac/Simpilirix™ vaccine (gD-2+alum+MPL), would prevent genital herpes in HSV-1 seronegative women. On September 30, 2010, the disappointing results were announced; immunization with a gD-2 subunit vaccine did not reduce the rate at which women acquired HSV-2 genital herpes [49]. These results have left many of the world's foremost experts questioning how an immunogen as potent as the gD-2 subunit vaccine could fail to prevent genital herpes [48].

Theoretical considerations suggest that a gD-2 subunit vaccine may not elicit the maximum protection against HSV-2 that is attainable. Ideally, a HSV-2 vaccine would drive clonal expansion of a broad repertoire of HSV-2-specific B and T cells, and this diverse subpopulation of lymphocytes would confer complete resistance to HSV-2 infection. Given that gD-2 is only 1 of 80 HSV-2 proteins, it is unlikely that immunization with gD-2 may drive clonal expansion of the body's full repertoire of HSV-2-specific B and T cells. Thus, we have questioned whether a live-attenuated HSV-2 virus would better prepare the body's immune system to resist HSV-2 infection [50].

Earlier studies of HSV-1 *ICP0* ^−^ mutant viruses led us to propose that mutagenesis of HSV-2's *ICP0* gene might yield viruses that were interferon-sensitive, avirulent, and suitable for use as a live HSV-2 vaccine strain [50], [51]. We have recently corroborated the validity of these predictions, and identified a tenable HSV-2 *ICP0* ^−^ vaccine strain, HSV-2 0ΔNLS [52].

Using this novel reagent, the current study was initiated to determine if mice immunized with the live HSV-2 0ΔNLS virus were more resistant to HSV-2 genital herpes than mice immunized with a gD-2 subunit vaccine. Consistent with previous reports [6], [37], [38], mice immunized with gD-2_306t_, alum, and MPL mounted a very high antibody response against gD-2. However, only 3 of 45 gD-2-immunized mice survived vaginal or ocular challenge with an overwhelming dose of wild-type HSV-2 MS strain. In contrast, nearly 100% of mice immunized with the HSV-2 *ICP0* ^−^ virus, 0ΔNLS, survived the same rigorous challenges. Importantly, 0ΔNLS-immunized mice were as resistant to superinfection with wild-type HSV-2 as mice that recovered from a primary infection with wild-type HSV-2. We present three lines of evidence that mice immunized with the 0ΔNLS virus were 10 to 100 times better protected against HSV-2 genital herpes than mice immunized with a gD-2 subunit vaccine, similar in composition to the Simpilirix™ vaccine [48], [49]. These results suggest that a HSV-2 vaccine might be more likely to prevent genital herpes if it contained a live-attenuated HSV-2 virus rather than a single HSV-2 protein [41], [48], [53].

## Results

### HSV-2 0ΔNLS is avirulent and immunogenic

HSV-2 0ΔNLS is a live-attenuated virus that is avirulent in mice following inoculation of the eyes [52]. A test was conducted to determine if HSV-2 0ΔNLS was avirulent and immunogenic when administered to mice by mucosal or subcutaneous routes of vaccination; namely, the nostrils (mucosal route), rear footpads (subcutaneous route), or vagina (mucosal route). Wild-type HSV-2 MS (*ICP0* ^+^) virus was included as a control to verify that the 0ΔNLS mutation was necessary to attenuate the pathogenesis of HSV-2 infection.

**Figure 1:**
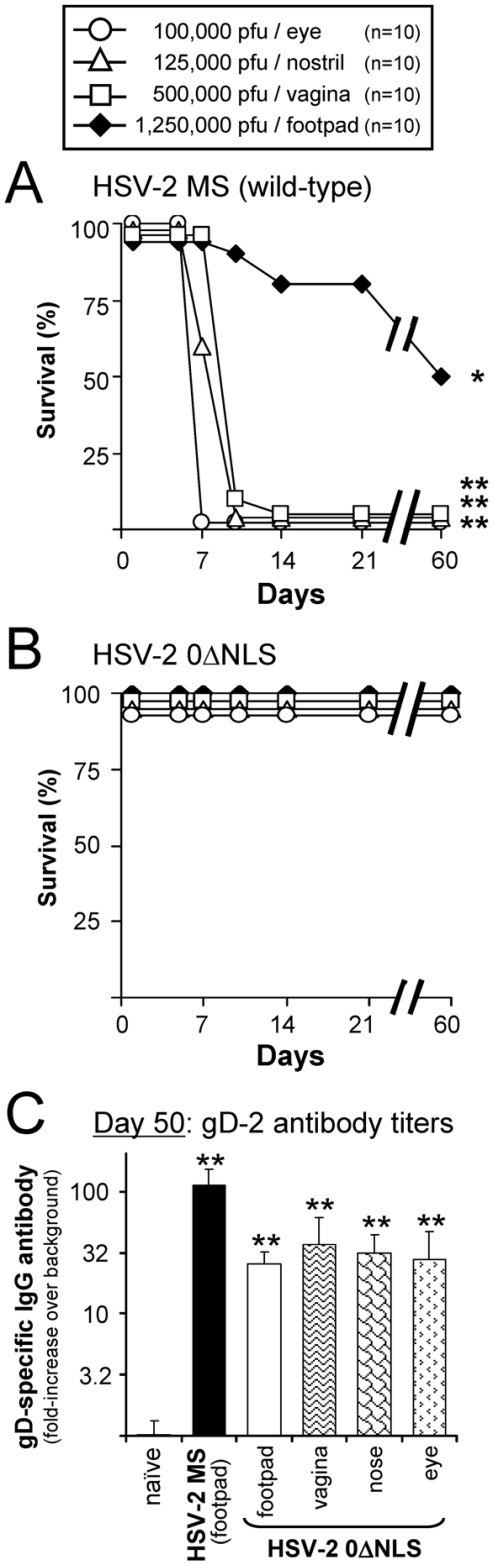
HSV-2 0ΔNLS is avirulent and immunogenic in female ICR mice. Duration of survival following inoculation of naïve mice with culture medium containing 25,000 pfu per µl of (**A**) HSV-2 MS or (**B**) HSV-2 0ΔNLS following placement of 4 µl on left and right scarified eyes; 5 µl in left and right nostrils; 50 µl in left and right, rear footpads; or 20 µl instilled into the vaginal vault (n = 10 mice per group). A single asterisk (*) denotes a probability, p, <0.05 and a double asterisk (**) denotes p<0.001 that matched pairs of mice inoculated with (A) HSV-2 MS or (B) HSV-2 0ΔNLS survived at equivalent frequencies, as calculated by Fisher's Exact Test. (**C**) Mean ± sem abundance of gD-2 specific IgG antibody in mouse serum on Day 50 p.i., as determined by ELISA on 1∶100 dilutions of mouse serum (n = 10 per 0ΔNLS-immunization group; n = 5 MS-immunized mice). The y-axis represents relative units of IgG abundance expressed as “fold-increase above background,” as determined relative to a 0.33-log dilution series of high titer anti-HSV-2 antiserum that provided the standard curve that defined the quantitative relationship between anti-gD-2 IgG antibody abundance and the colorimetric development in each well of the ELISA plate (i.e., the standard curve had a goodness-of-fit of r^2^ = 0.99). A double asterisk (**) denotes a probability, p, <0.001 that gD-2-antibody levels were equivalent to naïve mice, as determined by one-way ANOVA and Tukey's post hoc t-test.

Groups of n = 10 female ICR mice were inoculated with HSV-2 MS or HSV-2 0ΔNLS by the following routes: ***i.*** 100,000 pfu per eye; ***ii.*** 125,000 pfu per nostril; ***iii.*** 500,000 pfu per vagina; or ***iv.*** 1,250,000 pfu per rear footpad. Ocular and vaginal swabs confirmed that HSV-2 MS and 0ΔNLS consistently replicated in mice (Fig. S1). HSV-2 MS produced fatal encephalitis in 100% of mice inoculated in the eyes between Days 6 and 7 post-inoculation (p.i.), and all intranasally-inoculated mice succumbed by Day 9 p.i. (Fig. 1A). HSV-2 0ΔNLS did not produce any overt disease that was evident upon visual inspection of mice inoculated in the eyes or nostrils (Fig. 1B). HSV-2 MS inoculation of the vagina of medoxyprogesterone-treated mice produced lethal disease between Days 8 and 12 p.i., and death coincided with the onset of hindlimb paralysis (Fig. 1A). HSV-2 0ΔNLS did not produce any overt disease in mice inoculated vaginally (Fig. 1B). Mice inoculated with HSV-2 MS in the rear footpads were slow to develop lethal disease, and 50% survived until Day 60 p.i. (Fig. 1A). HSV-2 0ΔNLS did not produce any overt disease in mice inoculated in the rear footpads (Fig. 1B). Thus, the 0ΔNLS mutation in the *ICP0* gene allowed a mild HSV-2 infection to be established in ICR mice that did not produce any overt symptoms of disease following inoculation of the eyes, nose, vagina, or footpads. However, histological analysis was not performed on HSV-2 0ΔNLS-infected tissues, and we cannot exclude the possibility that HSV-2 0ΔNLS infection caused pathological changes that were not visible upon gross examination of 0ΔNLS-inoculated mice.

HSV-specific IgG antibody levels were compared amongst 0ΔNLS-inoculated mice and mice surviving footpad inoculation with HSV-2 MS. Mice were bled on Day 50 p.i., and sera were tested for the presence of gD-2-specific IgG antibody [34]. MS-footpad-inoculated mice possessed gD-2 antibody levels that were an average 100-fold above the background of ELISA (Fig. 1C). All HSV-2 0ΔNLS-immunized mice possessed gD-2 antibody levels that were 25- to 32-fold above background (Fig. 1C). Thus, regardless of whether mice were inoculated in the eyes, nose, feet, or vagina, immunization with the live-attenuated HSV-2 0ΔNLS virus elicited a significant IgG antibody response directed against HSV-2's entry receptor, gD-2.

### HSV-2 0ΔNLS-immunized mice acquire immunity to wild-type HSV-2

**Figure 2:**
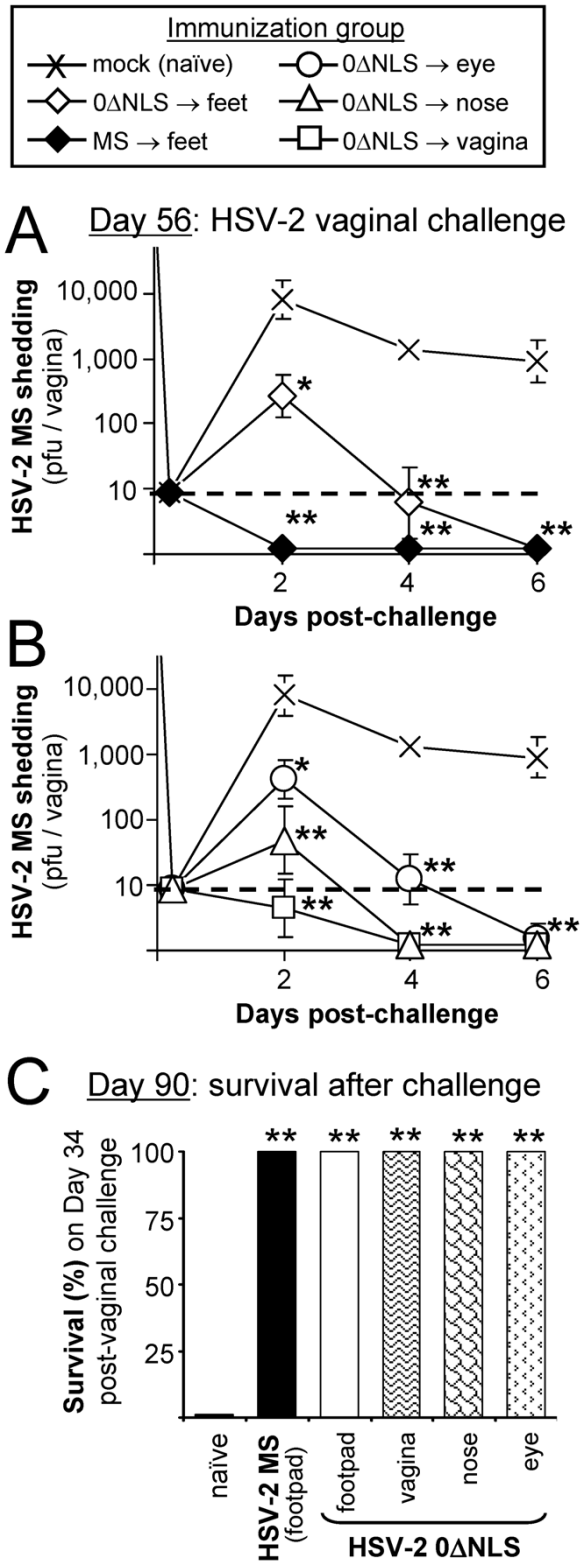
Mice immunized with HSV-2 0ΔNLS are resistant to HSV-2 vaginal challenge. Mice were treated with 2 mg medoxyprogesterone 7 and 3 days prior to vaginal HSV-2 challenge [54]. On Day 56 p.i., HSV-2 0ΔNLS- and MS-immunized mice were challenged with 500,000 pfu per vagina of HSV-2 MS. (**A**) HSV-2 shedding from the vagina between Days 2 and 6 post-challenge in naïve mice (n = 10) versus mice inoculated in the rear footpads with HSV-2 MS (n = 5) or HSV-2 0ΔNLS (n = 5). (**B**) HSV-2 shedding from the vagina of naïve mice versus mice inoculated in the eyes, nose, or vagina with HSV-2 0ΔNLS (n = 5 per group). In panels A and B, a single asterisk (*) denotes a probability, p, <0.05 and a double asterisk (**) denotes p<0.001 that HSV-2 shedding was equivalent to naïve controls on that day, as determined by one-way ANOVA and Tukey's post hoc t-test. (**C**) Survival frequency of naïve mice (n = 10) versus immunized mice (n = 5 per group) after HSV-2 challenge of the vagina. A double asterisk (**) denotes p<0.001 that survival frequency was equivalent to naïve mice.

On Day 56 p.i., protective immunity was assessed in the MS- and 0ΔNLS-immunized mice described above. In this and all vaginal challenges, a robust HSV-2 infection was established by ***1.*** treating mice with 2 mg medoxyprogesterone 7 and 3 days prior to challenge [54], [55], and by ***2.*** inoculating mice with 500,000 pfu per vagina of wild-type HSV-2 MS. On Days 2, 4, and 6 post-challenge, naïve mice shed an average 7900, 1300, and 900 pfu per vagina, respectively (X symbols in Figs. 2A, 2B). In contrast, MS-footpad-immunized mice did not shed detectable levels of HSV-2 challenge virus from their vaginas on Days 2, 4, or 6 post-challenge (⧫ symbols in Fig. 2A). Likewise, 0ΔNLS-footpad-immunized mice shed an average 30- and 220-fold less HSV-2 challenge virus than naïve mice on Days 2 and 4 (⋄ symbols in Fig. 2A). On Days 4 and 6 post-challenge, shedding of HSV-2 per vagina was reduced by >100-fold in all 0ΔNLS-immunized mice relative to naïve controls, regardless of the specific route of immunization (Fig. 2A, 2B). Likewise, all 0ΔNLS-immunized mice survived HSV-2 vaginal challenge regardless of the route of immunization, whereas 0 of 10 naïve mice survived HSV-2 vaginal challenge (Fig. 2C).

Mice immunized with HSV-2 0ΔNLS were also challenged on Day 56 with 100,000 pfu per eye of wild-type HSV-2 (Fig. S2). Consistent with the results of vaginal challenge, 0ΔNLS-immunized mice shed significantly less HSV-2 challenge virus per eye than naïve mice on Days 1 and 2 (Fig. S2A, S2B). Likewise, while 0 of 10 naïve mice survived HSV-2 MS challenge of the eyes, all 0ΔNLS-immunized mice survived ocular HSV-2 challenge regardless of the route of 0ΔNLS immunization (Fig. S2C). Therefore, mice immunized with HSV-2 0ΔNLS possessed potent and systemic protection against wild-type HSV-2.

### Footpad immunization with HSV-2 0ΔNLS versus a gD-2 subunit vaccine

**Figure 3:**
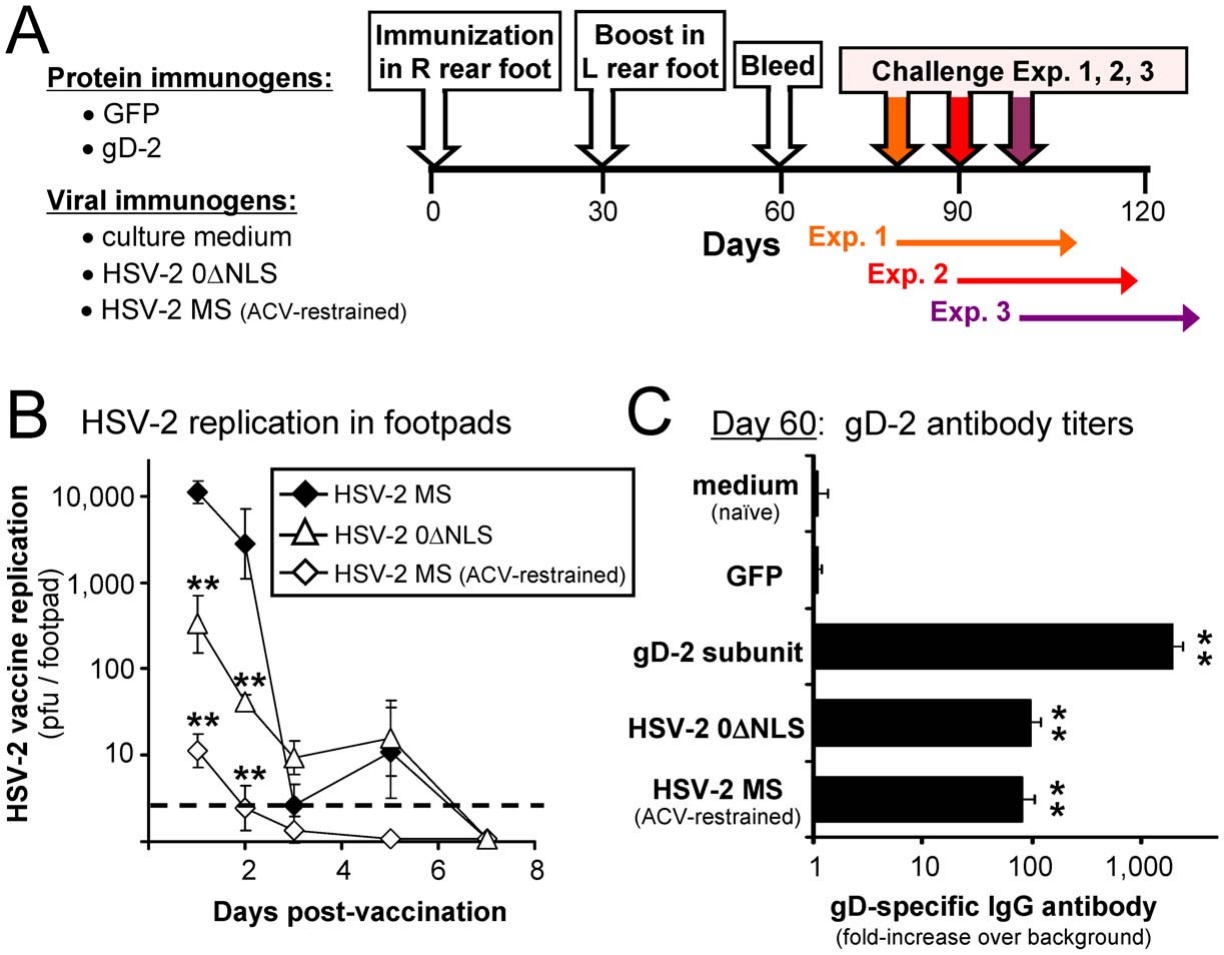
Immunization with HSV-2 0ΔNLS, gD-2, or control immunogens. (**A**) Design of vaccine-challenge experiments. Protein-immunized mice were injected in their right, rear footpads on Day 0 with 10 µg monophosphoryl lipid A, 2.5 µg gD-2 or GFP, and alum (n = 40 per group). On Day 30, mice received an equivalent immunization in their left, rear footpads. Virus-immunized mice received injections on Days 0 and 30 of culture medium (mock), 1×10^6^ pfu of HSV-2 0ΔNLS, or 1×10^6^ pfu of HSV-2 MS (n = 40 per group). Mice immunized with HSV-2 MS received 1 mg/ml acyclovir in drinking water from Days −1 to +20 p.i. On Day 60, blood was harvested from all mice, and on Days 80, 90, or 100, mice were challenged with wild-type HSV-2 MS. (**B**) HSV-2 replication in mouse footpads. In a parallel experiment, mice were footpad-injected with 1×10^6^ pfu of HSV-2 MS in the presence or absence of oral acyclovir (ACV) or 1×10^6^ pfu of HSV-2 0ΔNLS. On Days 1, 2, and 3 p.i., footpad titers of infectious HSV-2 were determined in n = 8 mice per group; on days 5 and 7 p.i., footpad titers were determined in n = 4 mice per group. All datum points represent mean ± sem pfu per footpad. A double asterisk (**) denotes p<0.001 that viral titers per footpad were the same as HSV-2 MS-inoculated mice not treated with acyclovir. (**C**) Mean ± sem relative abundance of gD-2 specific IgG antibody in mouse serum on Day 60 p.i., as determined by ELISA on 1∶100 dilutions of mouse serum (n = 30 per group). Relative units of IgG abundance are expressed as “fold-increase above background,” as determined relative to a 0.33-log dilution series of high titer anti-HSV-2 antiserum that provided the standard curve that defined the quantitative relationship between anti-gD-2 IgG antibody abundance and colorimetric development. A double asterisk (**) denotes p<0.001 that gD-2-antibody levels were equivalent to naïve (medium-treated) mice, as determined by one-way ANOVA and Tukey's post hoc t-test.

The efficacy of HSV-2 0ΔNLS-induced protection against genital herpes was compared to a gD-2 subunit vaccine, similar in composition to Glaxo Smith Kline's Simpilirix™ vaccine [6], [38], [48]. Groups of n = 40 mice were immunized on Day 0 in their right, rear footpads with: ***1.*** 2.5 µg gD-2, alum, and 10 µg monophosphoryl lipid A (MPL); ***2.*** 2.5 µg GFP control antigen, alum, and 10 µg MPL; ***3.*** culture medium (vehicle); ***4.*** 1×10^6^ pfu of HSV-2 0ΔNLS; or ***5.*** 1×10^6^ pfu of wild-type HSV-2 MS (Fig. 3A). Mice inoculated in their rear footpads with wild-type HSV-2 MS were given 1 mg per ml acyclovir (ACV) in their drinking water between Days −1 and +20 p.i. to limit the pathogenesis of the primary infection. Consequently, 100% of ACV-treated mice survived HSV-2 MS inoculation of the right, rear footpad (Fig. 3A).

Replication of HSV-2 MS or 0ΔNLS in the footpads of vaccine recipients was verified in parallel groups of mice, as follows. In the absence of ACV, HSV-2 MS replicated to peak titers of 1,000 to 10,000 pfu per footpad on Days 1 and 2 p.i., and viral replication was low to undetectable thereafter (Fig. 3B). HSV-2 0ΔNLS replicated to respective titers of ∼300 and 50 pfu per footpad on Days 1 and 2 p.i., which was 30-fold lower than wild-type HSV-2 (Fig. 3B). In ACV-treated mice, peak titers of HSV-2 MS replication were only ∼10 pfu per footpad, which was 1,000-fold lower than HSV-2 MS titers in untreated mice (Fig. 3B). This latter observation appeared to explain how 100% of ACV-treated mice survived HSV-2 MS inoculation of the rear footpad without any overt signs of disease.

On Day 30, all mice were boosted via injection of the same immunogens into their left, rear footpads (Fig. 3A). Mice were bled on Day 60 p.i. and sera were analyzed for the presence of gD-2-specific antibody. Mice immunized with culture medium (naïve) or GFP did not possess detectable levels of gD-2-specific antibody (Fig. 3C). Mice vaccinated with the gD-2 subunit vaccine possessed titers of gD-2-antibody that were 2,000-fold above background (Fig. 3C). Based on the 1∶100 dilution of serum used in the ELISA, gD-2-vaccinated mice had an average gD-2 antibody titer of 1∶200,000. Mice vaccinated with HSV-2 0ΔNLS or MS possessed far lower levels of gD-2 antibody that were 97- and 85-fold above background, respectively (Fig. 3C). Thus, the gD-2 subunit vaccine formulation used in the current study elicited a gD-2 antibody response similar in potency to that described elsewhere [6], [37], [38].

### Protective immunity elicited by vaccination with HSV-2 0ΔNLS versus gD-2

**Figure 4:**
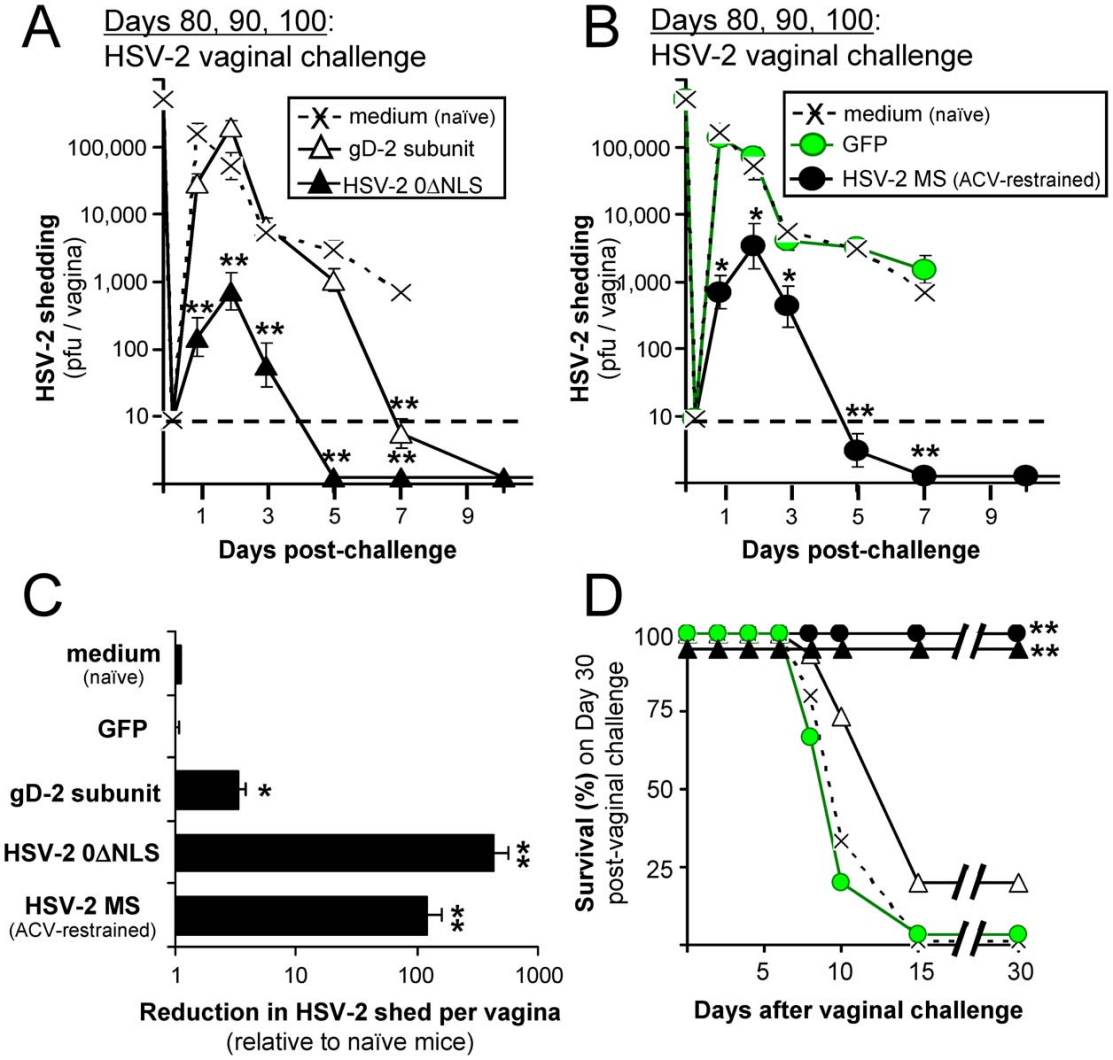
Resistance of naïve versus immunized mice to vaginal HSV-2 infection. Mice were treated with 2 mg medoxyprogesterone 7 and 3 days prior to vaginal HSV-2 challenge [54]. On Days 80, 90, or 100 p.i., mice were challenged with 500,000 pfu per vagina of HSV-2 MS (n = 5 per group). The summated results from all three experiments are presented in each panel (∑n = 15 per group). (**A**) Vaginal HSV-2 shedding between Days 1 and 7 post-challenge in mice that were naïve or immunized with gD-2_1-306t_ versus HSV-2 0ΔNLS. (**B**) Vaginal HSV-2 shedding in mice that were naïve or immunized with GFP versus HSV-2 MS. In panels A and B, a single asterisk (*) denotes p<0.05 and a double asterisk (**) denotes p<0.001 that HSV-2 shedding was equivalent to naïve mice on that day, as determined by one-way ANOVA and Tukey's post hoc t-test. (**C**) Mean ± sem reduction in HSV-2 shedding on Days 1–7 post-challenge relative to the average titer of HSV-2 shed by naïve mice on that day (n = 75 per group). In panel C, a single asterisk (*) denotes p<0.05 and a double asterisk (**) denotes p<0.001 that reductions in vaginal shedding of HSV-2 MS were significantly greater than a value of 1, as determined by one-way ANOVA and Tukey's post hoc t-test. The difference in reductions in HSV-2 MS vaginal shedding between 0ΔNLS- and gD-2-immunized mice was significant (p<10^−23^; two-sided, paired t-test). (**D**) Survival frequency over time following HSV-2 MS challenge of the vagina. A double asterisk (**) denotes p<0.001 that survival frequency was equivalent to naïve mice, as determined by Fisher's Exact Test. The survival rate of gD-2 immunized mice was not significantly different than naïve mice (p = 0.22, Fisher's Exact Test).

On Days 80, 90, and 100 post-immunization, n = 5 mice per group were challenged with 500,000 pfu per vagina of wild-type HSV-2 (Fig. 3A). The summated results of replicate challenge experiments are presented, as follows. On Days 1 and 2 post-challenge, naïve mice shed an average 200,000 and 50,000 pfu per vagina, respectively (‘X’ symbols in Figs. 4A, 4B). Immunization with gD-2 or GFP did not alter the course of HSV-2 vaginal shedding for the first 5 days post-challenge (Fig. 4A, 4B). In contrast, immunization with HSV-2 0ΔNLS or MS reduced shedding of the HSV-2 challenge virus from the vagina at all times (Fig. 4A, 4B). On average, 0ΔNLS- and MS-immunized mice shed a respective 430- and 120-fold less HSV-2 challenge virus between Days 1 and 7 relative to naïve mice (Fig. 4C). In contrast, gD-2 and GFP-immunized mice shed a respective 3.4- and 0.9-fold less HSV-2 per vagina relative to naïve mice (Fig. 4C). Thus, mice immunized with HSV-2 0ΔNLS shed an average 125-fold less HSV-2 MS from their vaginas relative to gD-2-immunized mice, and this difference was significant (p<10^−23^; two-sided, paired t-test).

None of the naïve or GFP-immunized mice survived HSV-2 vaginal challenge, and only 20% of gD-2-immunized mice survived the same challenge (Fig. 4D). In contrast, 15 of 15 0ΔNLS-immunized mice and 15 of 15 MS-immunized mice survived HSV-2 vaginal challenge (Fig. 4D). Most 0ΔNLS- and MS-immunized mice survived without any overt symptoms of disease for 30 days after vaginal challenge; however, 1 of 15 mice in each group exhibited limited perivaginal fur loss between 10 and 30 days after HSV-2 vaginal challenge.

To verify that protective immunity against HSV-2 was not unique to the vagina, mice were also challenged with 100,000 pfu per eye of HSV-2 MS on Days 80, 90, and 100 post-vaccination. In general, equivalent results were obtained in ocular and vaginal challenge experiments (Figs. 4 and S3). However, fewer mice survived HSV-2 MS challenge of the eyes because of the extraordinary rapidity with which HSV-2 MS spreads from the eyes to the central nervous system (6–7 days to death; Fig. 1A). Consequently, 0 of 15 mice immunized with culture medium, GFP, or gD-2 survived HSV-2 MS challenge of the eyes (Fig. S3D). In contrast, 14 of 15 mice immunized with HSV-2 0ΔNLS- or MS survived the same, stringent ocular HSV-2 challenge (Fig. S3D), and ∼90% of these mice survived without any overt symptoms of disease for 30 days after challenge. However, histological analysis was not performed on HSV-2 MS-challenged vaginas or eyes, and we cannot exclude the possibility that HSV-2 MS caused pathological changes in the infected tissues that were not visible upon gross examination of mice. These results indicated that mice immunized with HSV-2 0ΔNLS were significantly better protected against wild-type HSV-2 challenge than gD-2-immunized mice, which remained vulnerable to wild-type HSV-2 replication and disease.

### Re-evaluating the magnitude of the HSV-2-specific IgG antibody response

Protective immunity against HSV-2 normally involves lymphocyte recognition of many HSV-2 proteins [56], [57], [58], [59]. Thus, we suspected that the gD-2 antibody-capture ELISA (Fig. 3C) was not representative of the magnitude of the *polyclonal* antibody response elicited by HSV-2 0ΔNLS or MS. To test this hypothesis, Day 60 serum samples collected from mice prior to HSV-2 challenge were re-analyzed in assays better suited to estimate the magnitude of a polyclonal antibody response against multiple HSV-2 antigens; namely, ***1.*** antibody-dependent neutralization of HSV-2 virions (which contain at least 6 glycoproteins; [43], [60]) and ***2.*** antibody-binding to HSV-2-infected Vero cells.

**Figure 5:**
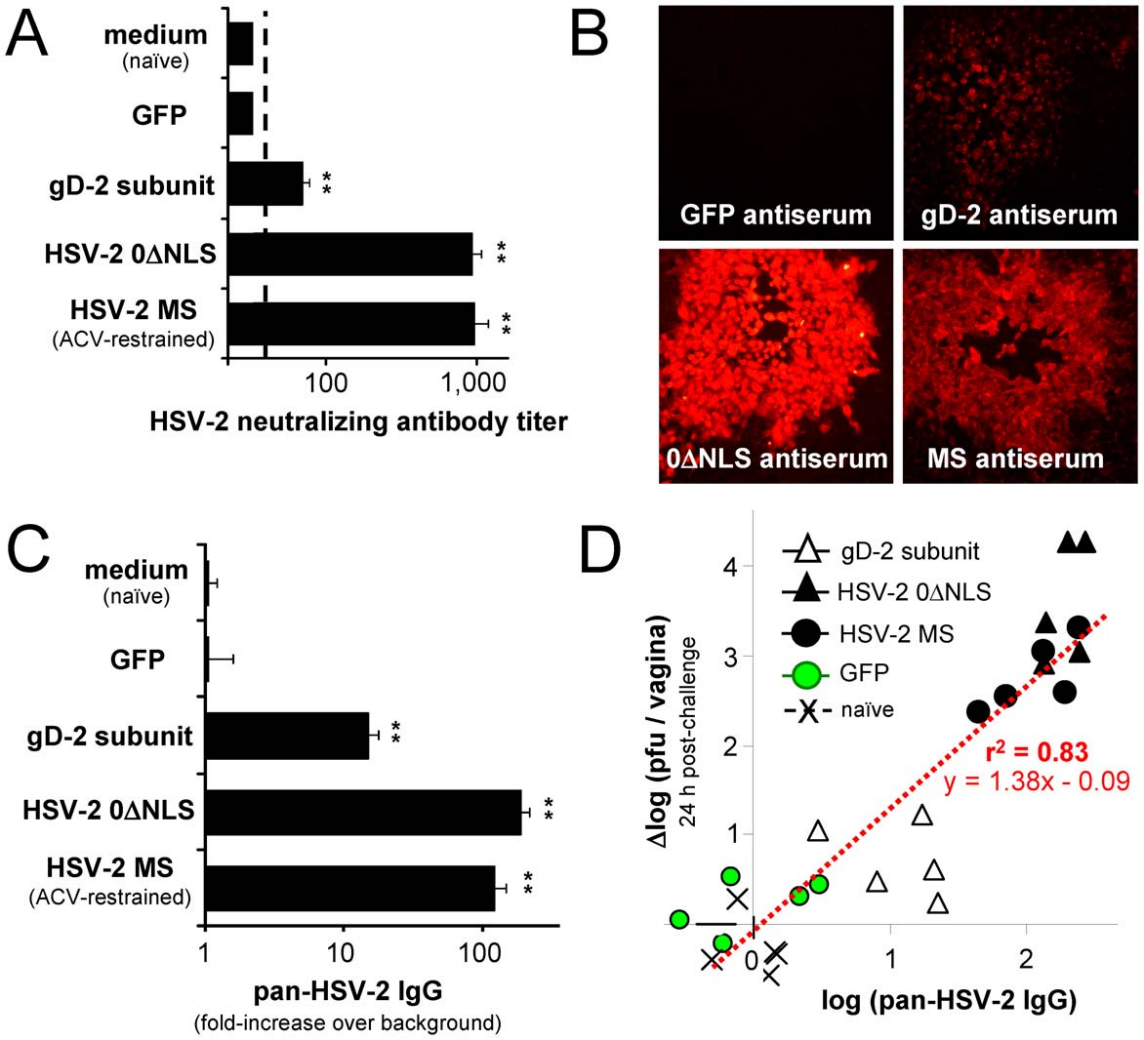
Polyclonal HSV-2 IgG antibody response elicited by HSV-2 0ΔNLS, gD-2, or control immunogens. (**A**) Mean ± sem neutralizing antibody titer of Day 60 serum samples (n = 20 per group). The titer of each serum sample was considered to be the reciprocal of the largest serum dilution that reduced HSV-2's cytopathic effect in Vero cell monolayers by at least 50%. (**B**) Representative immunofluorescent labeling of fixed HSV-2 plaques with a 1∶5,000 dilution of Day 60 serum from each immunization group. (**C**) Flow cytometric measurement of pan-HSV-2-specific IgG levels in Day 60 sera, as determined by IgG binding to fixed HSV-2-infected cells versus uninfected Vero cells (n = 8 per group). In panels A and C, a double asterisk (**) denotes p<0.001 that neutralizing antibody titers or pan-HSV-2 IgG levels were equivalent to naïve mice, as determined by one-way ANOVA and Tukey's post hoc t-test. The difference in pan HSV-2 IgG levels between 0ΔNLS- and gD-2-immunized mice was significant (p<0.0001; two-sided, paired t-test). (**D**) Regression analysis of the logarithm of pan-HSV-2 IgG levels (x-variable, as measured on Day 60) in n = 25 mice versus the logarithmic reduction in vaginal HSV-2 MS shedding (y-variable, as measured on Day 81) observed in the same n = 25 mice at 24 hours post-vaginal challenge. The x-variable data is based on a subset of the data summarized in Figure 5C, and likewise the y-variable data is based on a subset of the data summarized in Figure 4C. The individual datum points are derived from n = 5 mice per group that were immunized with medium (naïve), GFP, gD-2, HSV-2 0ΔNLS, or HSV-2 MS (ACV-restrained infection), as indicated in the legend in Panel D. The quantity on the y-axis, Δlog (pfu/vagina), represents the logarithmic decrease of HSV-2 MS shed from an individual mouse vagina at 24 hours post-challenge relative to 5.20 logs, which was the average titer of HSV-2 MS shed by naïve mice at 24 hours post-challenge. The goodness-of-fit of the correlation between log (pan-HSV-2 IgG) and Δlog (pfu/vagina) was r^2^ = 0.83 and the slope of the correlation was 1.38±0.13 (p<10^−9^).

Antisera from naïve- and GFP-immunized mice consistently failed to neutralize the infectivity of ∼175 pfu of HSV-2 at a serum dilution of 1∶46 (Fig. 5A). Antisera from gD-2-immunized mice had an average neutralizing antibody titer of 70 (Fig. 5A). Antisera from 0ΔNLS- or MS-immunized mice was ∼10-fold more potent in its capacity to neutralize infectious HSV-2. Specifically, antisera from mice immunized with HSV-2 0ΔNLS or MS had an average neutralizing antibody titer of ∼950 (Fig. 5A).

Immunofluorescence-based assays were developed to detect polyclonal antibody reactivity against the many HSV-2 proteins present in virus-infected cells. In the first assay, fixed and permeabilized HSV-2 plaques were incubated with 1∶5,000 dilutions of mouse serum, and stained with Alexa Fluor-594-conjugated secondary antibody. When fixed monolayers containing HSV-2 plaques were incubated with GFP-antiserum, binding of IgG to HSV-2 infected cells was not observed (Fig. 5B). Antisera from gD-2-immunized mice contained IgG that bound HSV-2 plaques, but the immunofluorescent signal was weak (Fig. 5B). In contrast, antisera from 0ΔNLS- or MS-immunized mice contained IgG that bound HSV-2-infected cells to much higher levels than surrounding, uninfected Vero cells (Fig. 5B).

Binding of IgG antibody to HSV-2-infected cells was quantified by a flow cytometry-based assay, which is summarized in Fig. S4. As expected, IgG antibodies in naïve serum and GFP-antiserum did not preferentially bind HSV-2-infected Vero cells versus uninfected Vero cells (Fig. 5C). Serum samples from gD-2-immunized mice contained IgG antibodies that bound HSV-2-infected cells to levels that were an average 15-fold greater than the background rate of antibody adhesion to uninfected cells (Fig. 5C). Serum from 0ΔNLS- or MS-immunized mice contained ∼10-fold higher levels of IgG antibody against total HSV-2 antigen. Specifically, 0ΔNLS antiserum contained pan-HSV-2 IgG levels that were an average 190-fold above background (Fig. 5C), and which were significantly greater than observed in gD-2-immunized mice (p<0.0001; two-sided, paired t-test).

The average pan-HSV-2 IgG level in each treatment group (Fig. 5C) appeared to correlate with reductions in vaginal HSV-2 MS shedding (Fig. 4C). To test the validity of this inference, regression analysis was applied to a data set collected from n = 25 mice (n = 5 per immunization group) included in both assays. Specifically, regression analysis was used to determine if log (pan-HSV-2 IgG level) in an individual mouse was predictive of log (reduction in HSV-2 MS shedding) observed in the same mouse 24 hours after vaginal challenge (Fig. 5D). Each mouse was challenged with HSV-2 MS on Day 80, and the reduction in HSV-2 MS vaginal shedding from an individual mouse was calculated as y = Δlog (pfu/vagina) = 5.20 log_10_ pfu/vagina (mean naïve titer) – HSV-2 titer shed from this mouse (Fig. 5D). Mice immunized with HSV-2 0ΔNLS or MS possessed the highest levels of pan-HSV-2 IgG (x-variable) and exhibited the largest reductions in HSV-2 MS vaginal shedding at 24 hours post-challenge (y-variable; Fig. 5D). Mice immunized with gD-2 possessed modest levels of pan-HSV-2 IgG and likewise exhibited modest reductions in HSV-2 MS vaginal shedding (Fig. 5D). Regression analysis confirmed that log (pan-HSV-2 IgG) in mice was predictive of the logarithmic reduction in HSV-2 MS vaginal shedding (Fig. 5D; r^2^ = 0.83, p<10^−9^ that x- and y-variables were unrelated). These data indicated that ***1.*** immunization with HSV-2 0ΔNLS elicited a greater pan-HSV-2 IgG antibody response than a gD-2 subunit vaccine (Fig. 5C; p<0.0001; two-sided, paired t-test), and ***2.*** pan-HSV-2 IgG antibody levels were highly predictive of protection against HSV-2 MS vaginal challenge (Fig. 5D).

### In vivo imaging of vaccine-induced protection: tests with HSV-2 MS-luciferase

**Figure 6:**
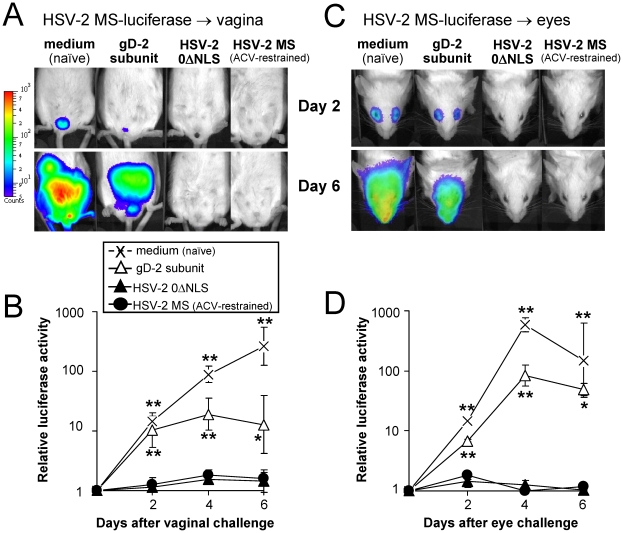
Vaccine-induced protection against HSV-2 MS-luciferase infection. (A and C) Mice were treated with 2 mg medoxyprogesterone 7 and 3 days prior to vaginal HSV-2 challenge [54]. On Day 130 p.i., mice were challenged with (**A**) 500,000 pfu per vagina or (**C**) 100,000 pfu per eye of HSV-2 MS-luciferase, and were anaesthetized and injected with 3 mg D-luciferin substrate at times post-challenge for imaging in a bioluminescent imager. Not shown in panels A or C are the age- and sex-matched, uninfected control mice included in these analyses that were anaesthetized and injected with 3 mg D-luciferin substrate at the same time, and which served as a background control to define the background level of light emission recorded from each mouse by the bioluminescent imager. (**B and D**) Mean ± sem of luciferase activity in mice challenged in the (**B**) vagina or (**D**) eyes with HSV-2 MS-luciferase, as measured by the fold-increase in light emission from each mouse relative to an uninfected background control mouse injected with 3 mg D-luciferin substrate. In the vaginally challenged group, each datum point represents the mean ± sem of luciferase activity based on ∑n = 5 per group (n = 3 challenged on Day 50 p.i. and n = 2 challenged on Day 130 p.i.). In the ocularly challenged group, each datum point represents the mean ± sem of luciferase activity based on ∑n = 4 per group (n = 2 challenged on Day 50 p.i. and n = 2 challenged on Day 130 p.i.). A single asterisk (*) denotes p<0.05 and a double asterisk (**) denotes p<0.001 that luciferase activity in HSV-2 MS-luciferase-challenged mice was significantly different from uninfected control mice injected with 3 mg D-luciferin, as determined by one-way ANOVA and Tukey's post hoc t-test. In both vaginal and ocular challenge tests, luciferase activity was significantly different between gD-2- and 0ΔNLS-immunized mice (p<0.0001; two-sided, paired t-test).

Individuals who carry latent HSV infections are resistant to superinfection with the same HSV serotype [61], [62], [63]. Likewise, mice immunized with HSV-2 0ΔNLS or MS might be resistant to superinfection with wild-type HSV-2 (Fig. 4). To test this hypothesis, HSV-2 challenge viruses were constructed whose spread could be imaged *in vivo*. Specifically, HSV-2 MS-luciferase and HSV-2 MS-GFP were constructed by inserting a luciferase or GFP expression cassette into HSV-2's non-essential *LAT* locus (Fig. S5). HSV-2 MS-luciferase spread was compared in immunized mice (Fig. 6), and the results are summarized as follows.

On Day 130 post-immunization, naïve or immunized mice were challenged with 500,000 pfu per vagina of HSV-2 MS-luciferase (n = 2 per group), and bioluminescent imaging was used to visualize viral spread (Fig. 6A). The spread of HSV-2 MS-luciferase from the vaginas of naïve and gD-2-immunized mice was readily visualized between Days 2 and 6 post-challenge (Fig. 6A). In contrast, luciferase expression was not detectable in 0ΔNLS- or MS-immunized mice (Fig. 6A). A replicate challenge experiment performed on Day 50 p.i. yielded equivalent results (n = 3 mice per group). The summated results were statistically analyzed, and the primary conclusions are presented (Fig. 6B). In HSV-2 MS-luciferase-challenged naïve mice, luciferase activity was an average 14-, 90-, and 270-fold above background on Days 2, 4, and 6 post-challenge, respectively (Fig. 6B). In gD-2-immunized mice, luciferase activity was reduced by 4.5- and 20-fold on Days 4 and 6 relative to naïve controls, respectively, but was still significantly above background (Fig. 6B). In contrast, luciferase activity in 0ΔNLS- or MS-immunized mice did not significantly differ from uninfected control mice injected with luciferin substrate (not shown) on Days 2, 4, or 6 post-challenge (Fig. 6B). Between Days 2 and 6 post-vaginal challenge, luciferase activity was an average 10-fold lower in 0ΔNLS- versus gD-2-immunized mice and this difference was significant (p<0.0001; two-sided, paired t-test).

HSV-2 MS-luciferase replication and spread may have occurred *inside* the vagina of 0ΔNLS- or MS-immunized mice at levels that were not detected by our bioluminescent imager. To address this caveat, an ocular challenge experiment was performed on Day 130 post-immunization such that an external surface served as the site of challenge (n = 2 mice per group). Following inoculation with 100,000 pfu per eye of HSV-2 MS-luciferase, luciferase activity was significantly greater than background in the eyes and faces of naïve and gD-2-immunized mice between Days 2 and 6 post-challenge (Fig. 6C, 6D). In contrast, luciferase activity was not detectable in mice immunized with HSV-2 0ΔNLS or MS (Fig. 6C, 6D). A replicate challenge experiment performed on Day 50 p.i. yielded equivalent results (n = 2 mice per group). The summated results were statistically analyzed, and the primary conclusions are presented (Fig. 6D). In HSV-2 MS-luciferase-infected naïve mice, luciferase activity in the eyes and faces of mice was an average 15-, 580-, and 150-times background on Days 2, 4, and 6 post-challenge, respectively (Fig. 6D). In gD-2-immunized mice, luciferase activity was reduced relative to naïve mice by 7- and 3-fold on Days 4 and 6 post-challenge, respectively (Fig. 6D). Between Days 2 and 6 post-ocular challenge, luciferase activity was an average 25-fold lower in 0ΔNLS-versus gD-2-immunized mice and this difference was significant (p<0.0001; two-sided, paired t-test). Therefore, mice immunized with HSV-2 0ΔNLS were 10- to 25-fold more resistant to HSV-2 MS-luciferase challenge than gD-2-immunized mice.

### In vivo imaging of vaccine-induced protection: tests with HSV-2 MS-GFP

**Figure 7:**
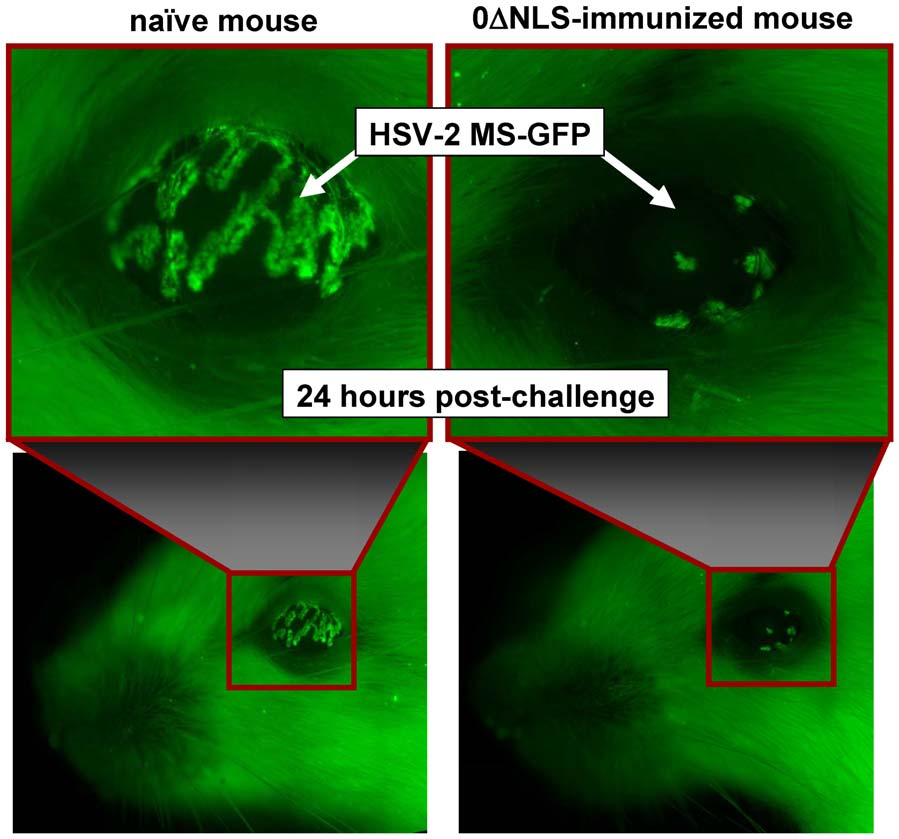
HSV-2 MS-GFP infection is established in the eyes of HSV-2 0ΔNLS-immunized mice, but is rapidly restricted. A naïve and HSV-2 0ΔNLS-immunized mouse, as observed 24 hours after challenge with 100,000 pfu per eye of HSV-2 MS-GFP. Experiments were performed on n = 3 mice per group and a representative animal is shown. The complete progression of HSV-2 MS-GFP infection in this naïve mouse versus immunized mouse is shown in Figure S6.

Bioluminescent imaging of luciferase activity *in vivo* is a macroscopic measurement. We reasoned that HSV-2 MS-luciferase might reproducibly establish microfoci of infection in 0ΔNLS- and MS-immunized mice that were not detectable with a bioluminescent imager. To address this possibility, naïve and 0ΔNLS-immunized mice were challenged with 100,000 pfu per eye of HSV-2 MS-GFP. At 24 hours post-challenge, GFP expression was imaged in mouse eyes and faces using an inverted fluorescent microscope (n = 3 per group). As predicted, foci of HSV-2 MS-GFP replication were consistently detected in the eyes of both naïve and 0ΔNLS-immunized mice at 24 hours post-challenge (Fig. 7). However, the extent of HSV-2 MS-GFP spread in the eyes (i.e., area of GFP expression) was restricted by an order of magnitude in 0ΔNLS-immunized mice relative to naïve controls (Fig. 7). Thus, 0ΔNLS-vaccine-induced protection against HSV-2 infection appeared to be active within just the first 24 hours post-challenge (Fig. 7). Consistent with this interpretation, HSV-2 MS-GFP consistently caused a zosteriform pattern of spread in naïve mice (Fig. S6), and fatal encephalitis developed within 8 days post-challenge. In contrast, sites of HSV-2 MS-GFP replication were not visible in the eyes or facial epithelium of 0ΔNLS-immunized mice at any time beyond Day 1 post-challenge (Fig. S6). Therefore, *in vivo* imaging indicated that 0ΔNLS-immunized mice were able to rapidly control the spread of a superinfecting HSV-2 virus (Fig. 6, 7, and S6).

### Summary of HSV-2 MS challenge experiments

In the current study, several hundred female ICR mice were challenged with the MS strain of wild-type HSV-2 in nineteen independent challenge experiments (Tables 1 and S1). The results of these tests are summarized, as follows. None of the naïve mice used in these tests survived HSV-2 MS challenge of the eyes (0 of 99) or vagina (0 of 25). None of the GFP-immunized mice survived HSV-2 MS challenge of the eyes (0 of 15) or vagina (0 of 15). None of the gD-2-immunized mice survived HSV-2 MS challenge of the eyes (0 of 30), but 3 of 15 survived HSV-2 MS challenge of the vagina (Table 1). Mice immunized with live HSV-2 viruses were far better protected against later exposures to wild-type HSV-2 MS. Specifically, 79 of 80 0ΔNLS-immunized mice survived HSV-2 MS challenge of the eyes, and 35 of 35 0ΔNLS-immunized mice survived HSV-2 MS challenge of the vagina (Table 1). Thus, 0ΔNLS-immunized mice were ∼15 times more likely to survive HSV-2 MS challenge than gD-2-immunized mice, and this difference was significant (Table 1; p<10^−8^, Fisher's Exact Test). Likewise, 46 of 49 MS-immunized mice survived HSV-2 MS challenge of the eyes, and 15 of 15 MS-immunized mice survived HSV-2 MS challenge of the vagina (Table 1).

**Table 1:** Survival rates in HSV-2 MS challenge experiments.

HSV-2 MS challenge of the eye (100,000 pfu/eye)					
	naïve^a^	GFP	gD-2	0ΔNLS	MS
Survival rate(# of experiments)	0/99^b^(n = 15)	0/15(n = 3)	0/30(n = 5)	79/80** ^,^ ‡(n = 11)	46/49**(n = 8)

HSV-2 MS challenge of the vagina (500,000 pfu/vagina)					
	Naïve	GFP	gD-2	0ΔNLS	MS
Survival rate(# of experiments)	0/25(n = 4)	0/15(n = 3)	3/15*(n = 3)	35/35** ^,^ ‡(n = 4)	15/15**(n = 3)

Summated results					
	Naïve	GFP	gD-2	0ΔNLS	MS
Survival rate	0/124(n = 19)	0/30(n = 6)	3/45*(n = 8)	114/115** ^,^ ‡(n = 15)	61/64**(n = 11)

a Immunization status of mice at the time of HSV-2 MS challenge, which were vaccinated 45 to 190 days earlier with culture medium (naïve), GFP, gD-2, 0ΔNLS, or HSV-2 MS.

b Frequency of mice that survived until 30 days after challenge with wild-type HSV-2 MS. The total number of independent experiments performed is indicated by the n-value in parentheses.

*p<0.05 that the survival frequency was equivalent to naïve mice following HSV-2 MS challenge, as determined by Fisher's Exact Test.

**p<10^−6^ that survival frequency was equivalent to naïve mice following HSV-2 MS challenge, as determined by Fisher's Exact Test.

‡ p<10^−8^ that survival frequency was equivalent between mice immunized with HSV-2 0ΔNLS versus a gD-2 subunit vaccine following HSV-2 MS challenge, as determined by Fisher's Exact Test.

**Figure 8:**
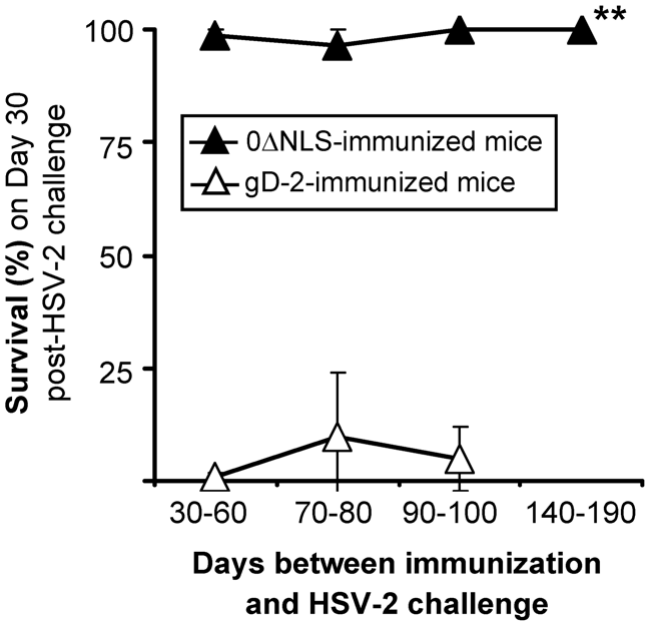
HSV-2 0ΔNLS-induced protective immunity does not decline between Days 30 and 190 post-immunization. The mean ± sem frequency of survival following HSV-2 MS challenge was compared over time in mice immunized with HSV-2 0ΔNLS or gD-2. The gD-2 plot is based on survival frequencies observed in challenge experiments performed between Days 30–60 (n = 1), 70–80 (n = 3), and 90–100 (n = 4) post-immunization. The 0ΔNLS plot is based on survival frequencies observed in challenge experiments performed between Days 30–60 (n = 5), 70–80 (n = 4), 90–100 (n = 4), and 140–190 (n = 2) post-immunization. Specific outcomes of the n = 15 challenge experiments are summarized in Table S1. The double asterisk (**) denotes that differences in percent survival of 0ΔNLS-versus gD-2-immunized mice following HSV-2 MS challenge were significant (p<10^−15^; two-sided Student's t-test).

Based on the results of individual challenge experiments (Table S1), we compared the survival rates of gD-2 and 0ΔNLS-immunized mice as a function of time between immunization and HSV-2 MS challenge (Fig. 8). In a total of 8 experiments, 8±6% of gD-2-immunized mice survived HSV-2 MS challenge (Fig. 8, Table S1). In a total of 15 experiments, 99±1% of 0ΔNLS-immunized mice survived HSV-2 MS challenge (Fig. 8, Table S1). This difference in survival frequency between 0ΔNLS- and gD-2-immunized mice was significant (p<10^−15^; two-sided Student's t-test). At all times post-immunization, gD-2 immunized mice were incompletely protected against HSV-2 MS (Fig. 8). In contrast, nearly 100% of 0ΔNLS-immunized mice survived HSV-2 MS challenge regardless of whether they were challenged on Days 56, 80, 100, or 190 post-immunization (Fig. 8). We conclude that mice immunized with a live viral vaccine, HSV-2 0ΔNLS, possessed significantly greater protection against lethal HSV-2 challenge than mice immunized with a gD-2 subunit vaccine.

## Discussion

### Measuring vaccine-induced resistance to HSV-2

HSV-2 vaccines are often described in the binary terms of *effective* versus *ineffective*. Such qualitative terms and qualitative measures of protection (e.g., reductions in disease and death) provide only a crude basis for estimating the potency of HSV-2 vaccine candidates. The quantity of *vaccine-induced resistance to HSV-2* is a function of the frequency of HSV-2 specific B and T cells that have encountered their cognate antigen, and as a result have proliferated and/or differentiated into memory or effector cells. The adaptive immune response to any antigen involves thousands to millions of lymphocytes, and thus behaves as a continuous variable that varies over at least 2 to 3 orders of magnitude. If adaptive immunity to HSV-2 behaves as a continuous variable in nature, then the potency of *vaccine-induced resistance to HSV-2* is best described in similarly quantitative terms.

Such theoretical considerations have little value unless a method exists to measure the proposed quantity of *vaccine-induced resistance to HSV-2*. Herein, we identify four measures that strongly correlate with functional resistance to HSV-2; namely, ***1.*** quantitative reductions in shedding of HSV-2 challenge virus from the vagina (Fig. 4C); ***2.*** abundance of IgG antibody against total HSV-2 antigen (Fig. 5C); ***3.*** *in vivo* imaging and quantitation of restricted spread of a bioluminescent HSV-2 challenge virus from the site of challenge (Fig. 6); and ***4.*** *in vivo* imaging of restricted spread of a GFP-expressing HSV-2 challenge virus at the site of challenge (Fig. 7). The last three of these methods are novel. We propose that such measures of *vaccine-induced resistance to HSV-2* provide a superior basis for analyzing HSV-2 vaccine potency, as opposed to more qualitative measures of vaccine-induced protection, such as ***i.*** reduced disease score or ***ii.*** increased survival.

### Resistance to HSV-2 elicited by a gD-2 subunit versus 0ΔNLS virus

Mice immunized with a gD-2 vaccine exhibited significant resistance to HSV-2 relative to naïve mice, as demonstrated by ***i.*** a 3.4-fold reduction in HSV-2 challenge virus shedding from the vagina (Fig. 4C); ***ii.*** a 15-fold increase in pan-HSV-2 IgG levels (Fig. 5C); and ***iii.*** a 5- to 20-fold reduction in the spread of a bioluminescent HSV-2 challenge virus (Fig. 6).

These results are consistent with published findings that a gD-2 vaccine elicits significant resistance to HSV-2 infection [6], [37], [38], [42]. However, the magnitude of gD-2 vaccine-induced resistance (3- to 20-fold) was dwarfed by the HSV-2 0ΔNLS vaccine, which elicited a 200- to 500-fold increase in resistance to HSV-2 infection (Fig. 4, 5, 6). Such a reference point is absent from most gD-2 vaccine-challenge studies, which focus on the difference between naïve and gD-2-immunized animals [6], [37], [38], [42]. A recent study reported that naïve guinea pigs and guinea pigs vaccinated with gD-2, alum, and MPL shed equivalent levels of HSV-2 challenge virus from their vaginas on Days 1, 2, and 4 post-challenge, (Fig. 1C of Ref. [6]). This is comparable to what we observed in mice (Fig. 4A). Thus, we concur with the prevailing view that gD-2 subunit vaccines elicit significant resistance to HSV-2 infection. However, we note that a live HSV-2 *ICP0* ^−^ virus, 0ΔNLS, elicits 10 to 100 times greater protection against genital herpes.

One caveat of the current study is that tests were performed in mice, and not the preferred guinea pig HSV-2 vaccine-challenge model. Studies are in progress to determine if HSV-2 0ΔNLS will be equally effective as a HSV-2 vaccine in guinea pigs. A second caveat of the study is that we did not use Glaxo Smith Kline's proprietary AS04 adjuvant system [38]. Thus, our gD-2 vaccine formulation may not elicit the same level of resistance to HSV-2 that may be obtained with a more potent combination of gD-2 and adjuvant [6], [38].

### Why does the 0ΔNLS vaccine elicit greater protection against HSV-2 infection?

Prior to the recent Simpilirix™ vaccine trials, two earlier permutations of a gD-2 subunit yielded equivocal results in human clinical trials [36], [41]. Based on the premise that a more potent adjuvant (alum+MPL) would increase gD-2's efficacy as a genital herpes vaccine [37], [38], the NIAID invested $27.6 million in the Herpevac/Simpilirix™ vaccine trial for women conducted between 2003 and 2009 [48]. Although the data remain to be published, Glaxo Smith Kline recently announced that this latest gD-2 subunit vaccine did not reduce the rate at which women acquired HSV-2 genital herpes [49].

After investing so much time and effort into gD-2 subunit vaccines [33], there is ample cause for dismay at the failure of a promising genital herpes vaccine [48]. However, we would suggest that the relevant question moving forward is this: *“Is it reasonable to expect that an immune response elicited against a single HSV-2 protein should render the body completely resistant to infection with an actual HSV-2 virus?”*

Viruses are highly evolved genetic elements whose complexity exceeds the sum of their proteins. As a result, the polyclonal immune response to HSV-2 is more complex than the immune response to a model antigen (i.e., HSV≠OVA) (reviewed in Ref. [50], [64]). For the immunologist, we offer three reasons that a gD-2 subunit vaccine might be ineffective as a genital herpes vaccine [48]. First, it is unlikely that the adaptive immune response to HSV-2 hinges upon lymphocytes that recognize a single HSV-2 protein. Second, the T-cell response to HSV-2 involves, at a minimum, CD8^+^ T cells specific for the viral proteins ICP0, ICP4, ICP6, virion protein 5, and virion components encoded by the UL25, UL46, UL47, and UL49 genes [56], [57]. Third, we present evidence that the B-cell response to HSV-2 cannot be directed solely against gD-2 (Fig. 3C vs 5C); a subsequent study will address the fact that 0ΔNLS-immunized mice possess serum antibodies against >15 HSV-2 proteins (unpublished data of W. Halford). While it is reasonable to assume that HSV-2 0ΔNLS elicits a broader T cell response against HSV-2 than a gD-2 subunit vaccine, further investigation will be required to test this hypothesis.

For the non-immunologist, we offer the following analogy to illustrate why we question the validity of the assumption that a gD-2 subunit vaccine should be sufficient to prevent genital herpes [33], [48]. In human terms, relying on a gD-2 subunit vaccine to prevent genital herpes is like trying to capture a criminal (HSV-2) in a city (the body) by releasing a photograph of the criminal's nose (one subunit). While the nose may be a distinguishing feature, a full portrait of the criminal would generate a larger population of informants (antibodies and T-cells) better able to guide police (leukocytes) to the criminal's location. Immunologists will not require this analogy to appreciate that a live HSV-2 virus possesses far more epitopes than a HSV-2 subunit vaccine. However, most viral vaccines continue to be based upon subunit vaccines that contain <10% of the epitopes encoded by a virus. Perhaps it is time that immunological breadth should play a larger role in vaccine design, as vaccines that contain >25% of a pathogen's proteins would be more likely to confer useful protection against a pathogen.

Two decades ago, it appeared that gD-2 subunit vaccines would be sufficient to prevent genital herpes in the human population [33]. Today, the available evidence raises questions about the viability of gD-2 subunits or any strategy that proposes to elicit 100% protection against HSV-2 by immunizing with 1% of HSV-2's proteome [11], [12], [13], [14], [15], [16], [17], [18], [19]. We propose that the methods described herein for quantifying and visualizing *vaccine-induced resistance to HSV-2* (Fig. 4, 5, 6, 7) should prove useful in the future for determining if any subunit vaccine is as effective as a live-attenuated HSV-2 virus, such as 0ΔNLS.

### Live, replicating viruses: our most successful mode of vaccinating against viral disease

Originally, the term ‘*vaccination*’ meant to inoculate a person with a less virulent virus (*vaccinia* virus) to elicit a cross-protective immune response against the smallpox virus [65], [66]. When we have emulated the original approach, and used replication-competent viruses as the immunogen, we have succeeded in preventing yellow fever, poliomyelitis, mumps, measles, rubella, chickenpox, and shingles [67], [68], [69], [70], [71], [72]. In the past 30 years, research into live-attenuated viral vaccines has been largely replaced with research into viral subunit vaccines.

The term subunit ‘vaccine’ implies that the use of a viral protein, in lieu of a live virus, is a minor modification of the original strategy. However, we are unaware of any studies that validate this assumption. To the best of our knowledge, this is the first study in which immunization with (1) a viral protein subunit was compared in side-by-side fashion to (2) a live-attenuated variant of the same virus. In this study, immunization with a gD-2 subunit vaccine elicited 1–10% of the resistance to HSV-2 that was attainable with a live virus. We will be interested to learn if this observation is unique to HSV-2 or applies to other viruses. In principle, one means to test this hypothesis would be to compare the efficacy of immunization with one of the live viruses used in childhood vaccines (i.e., varicella-zoster virus [VZV] Oka strain [68], measles virus Schwarz strain [69], mumps virus Jeryl Linn strain [70], or rubella virus Cendehill strain [71]) relative to immunization with a protein subunit derived from the same virus.

An important question that remains to be addressed is whether or not viral replication is essential for HSV-2 0ΔNLS to elicit potent and sustained resistance to HSV-2 (Fig. 8). Hence, studies are in progress to compare the efficacy of the HSV-2 0ΔNLS vaccine strain relative to a replication-defective HSV-2 virus, similar in principle to Sanofi Pasteur's lead HSV-2 vaccine candidate, ACAM-529 [23], [24], [25]. We conclude by noting that while live viruses, such as HSV-2 0ΔNLS, are the basis of ∼75% of our effective viral vaccines [67], [68], [69], [70], [71], [72], there is not a single vaccine in clinical use that contains a replication-defective virus.

### The relative risk of live-attenuated viral vaccines

Vaccines based on recombinant proteins are safe, but have been ineffective against herpes and AIDS [73], [74]. The cost of subunit vaccines that rarely succeed is staggering. Each year that herpes and AIDS vaccines fail means that ***a.*** another 20 million people will be newly infected with HSV-2; ***b.*** another 2 million people will be newly infected with human immunodeficiency virus (HIV); and ***c.*** public faith in vaccines will continue to erode. The original ‘vaccination’ approach [75], in which a weakened virus served as the immunogen, underlies most of our successes in preventing viral disease. Perhaps it is time to reconsider the *relative* risk of the approach.

Live viral vaccines have always posed a risk to human health. However, history suggests that the low risk of a well-designed, live-attenuated viral vaccine is many thousands of times preferable to the certainty of disease and/or death that occurs when a viral pathogen is allowed to circulate in the human population. All of the live-attenuated viral vaccines developed in the 20^th^ century, which remain in clinical use worldwide, are generally well tolerated and highly effective in preventing viral disease [76], [77], [78], [79].

Mutagenesis of key viral activators, such as ICP0 [80], [81], [82], [83], is a general strategy that may be used to obtain live-replicating viruses that are avirulent, but which retain the capacity to present the entire protein signature of a viral pathogen to the adaptive immune system [50], [52]. The live viral vaccines used in clinical practice today were developed between 1940 and 1975 [67], [68], [69], [70], [71], [72], and rely on single-nucleotide substitutions for their attenuated phenotype [84], [85]. We propose that genetic engineering could be applied to derive a 2^nd^ generation of live-attenuated viruses that are equally effective, but which are safer (more stable) due to large, in-frame deletions that are unable to spontaneously revert to the wild-type genetic code.

### Conclusion

Because α-herpesviruses establish life-long infections in neurons, it has been suggested that a live α-herpesvirus vaccine would be too dangerous for use in humans [23]. Such claims contradict the fact that >55 million people have been inoculated with the varicella-zoster virus (VZV) Oka strain, which like HSV-2 is a neurotropic herpesvirus [77]. While the VZV Oka strain may establish latent infections in human neurons and reactivate from the latent state [86], clinical experience suggests that the risks associated with this live VZV vaccine are far outweighed by the benefits of not leaving a population susceptible to the >90% risk of being infected with wild-type VZV [87]. If clinical experience with the VZV Oka strain is any indication, then the risks associated with a live HSV-2 0ΔNLS vaccine would be preferable to the current situation in which wild-type HSV-2 is carried by ∼1 billion people, and ∼20 million people are newly infected each year with wild-type, disease-causing strains of HSV-2.

It remains to be determined if HSV-2 *ICP0* ^−^ mutant viruses, such as HSV-2 0ΔNLS, establish latent infections in vaccine recipients. Likewise, many questions remain about this novel class of live, interferon-sensitive HSV-2 vaccine [52]. However, what is clear is that mice immunized with HSV-2 0ΔNLS were 10- to 100-fold better protected against genital herpes than mice immunized with a gD-2 subunit vaccine. Therefore, we conclude that a HSV-2 vaccine would be more likely to prevent genital herpes if it contained a live- and appropriately-attenuated HSV-2 virus rather than another iteration of HSV-2 protein and adjuvant.

## Materials and Methods

### Ethics Statement

Mice were handled in accordance with the National Institutes of Health Guide for the Care and Use of Laboratory Animals. This study was approved by the Southern Illinois University School of Medicine Laboratory Animal Care and Use Committee in August 2008, and was assigned Protocol Number #205-08-019. These protocols remain active and are associated with a grant for the “Development of an Effective Genital Herpes Vaccine” (R21 AI081072).

### Cells and viruses

Vero cells and U2OS cells were obtained from the American Type Culture Collection (Manassas, VA), and High Five™ insect cells were obtained from Invitrogen Corporation (Carlsbad, CA). The ICP0-complementing L7 cell line [88] was kindly provided by Neal Deluca (University of Pittsburgh). Cell lines were propagated in Dulbecco's Modified Eagle's medium supplemented with 5% fetal bovine serum and antibiotics. The HSV-2 recombinant viruses used in this study (HSV-2 0ΔNLS, MS-GFP, and MS-luciferase) were derivative of HSV-2 MS (American Type Culture Collection). HSV-2 viruses were propagated in U2OS cells at 34°C following inoculation with a multiplicity of infection of 0.01 pfu per cell. For both wild-type HSV-2 and HSV-2 *ICP0* ^−^ mutant viruses, viral stocks were generated that were concentrated 10-fold by ultracentrifugation to achieve a minimum titer of 3×10^7^ pfu/ml. An HSV-2 glycoprotein D-expressing baculovirus was used to purify the gD-2_306t_ protein [34], and was generously provided by Dr. Gary Cohen and Dr. Roslyn Eisenberg (University of Pennsylvania). The detailed methods used to construct and characterize HSV-2 recombinant viruses used in this study are provided in a recent publication [52].

### Footpad immunization of mice

Female ICR mice were obtained from Harlan Sprague Dawley (Indianapolis, IN), and were first immunized at 6- to 10-weeks of age. Prior to immunization, mice were anesthetized by i.p. administration of xylazine (7 mg/kg) and ketamine (100 mg/kg).

#### i. Protein subunit vaccines

GFP or gD-2_306t_ protein were purified from baculovirus vector-infected insect cells as described below. Protein subunit vaccines were prepared by combining purified gD-2 or GFP with an equal volume of Imject alum adjuvant (Thermo Scientific, Rockford, IL) to achieve a protein concentration of 50 ng per µl. Monophosphoryl lipid A (Avanti Polar Biolipids, Alabaster, AL) was added to a concentration of 200 ng per µl. After 1 hour, mice were injected in right, rear footpads with 50 µl of this formulation on Day 0 such that mice were immunized with 2.5 µg of gD-2 or GFP and 10 µg of monophosphoryl lipid A. These doses of gD-2 were modeled after the gD-2 vaccine-challenge studies of Bourne, et al. (2003, 2005) [37], [38]. Mice received an equivalent injection in left, rear footpads on Day 30.

#### ii. HSV-2 viral vaccines

Virus-immunized mice were treated on Days 0 and 30 as described above, but were immunized with 50 µl of culture medium containing nothing (mock), 1×10^6^ pfu of HSV-2 0ΔNLS, or 1×10^6^ pfu of HSV-2 MS. At the time of the first immunzaiton, mice immunized with HSV-2 MS received 1 mg per ml acyclovir in their drinking water from Days −1 to +20 p.i. to limit the pathogenesis of the primary infection.

### Inoculation of mice with HSV-2 in the eyes, nostrils, or vagina

Female ICR mice that received a vaginal inoculum of HSV-2 were pre-treated 7 and 3 days prior to inoculation with 2 mg medoxyprogesterone (Depo-Provera®, Pfizer Inc., New York), which increases the efficiency of vaginal infection [54]. Immediately prior to HSV-2 inoculation, mice were anesthetized by i.p. administration of xylazine (7 mg/kg) and ketamine (100 mg/kg). Ocular inoculation of mice was performed by scarifying the left and right corneas with a 26-gauge needle, blotting tear film from the eyes with tissue paper, and by placing 4 µl complete DMEM containing 25,000 pfu/µl of HSV-2 MS or HSV-2 0ΔNLS on each scarified eye. Nasal inoculation of mice was achieved by instilling 5 µl per nostril of the same 25,000 pfu/µl solution of HSV-2 MS or 0ΔNLS from a micropipettor. For vaginal inoculation, the vagina was cleared of mucus by briefly introducing the cotton end of a cotton-tipped applicator into the vagina. Upon removal of the cotton swab, a pipettor was used to deliver 20 µl complete DMEM containing 25,000 pfu/µl of virus into the vaginal vault.

### Measurement of infectious HSV-2 titers in footpads, ocular tearfilm, or vaginal mucosa

Viral titers in the footpads of mice were determined by sacrificing mice at the indicated time, cutting the footpad off the end of the limb into 0.5 ml complete DMEM, and homogenizing the tissue with a Pro 200 homogenizer (Pro Scientific, Oxford, CT). Viral titers were determined by a 12-well plate plaque assay on ICP0-complementing L7 cells cultured in complete DMEM containing 0.5% methlycellulose. Viral titers in ocular tear film or the vaginal secretions of mice were determined at times after inoculation by swabbing the eye with a cotton-tipped applicator or inserting a cotton-tipped applicator into the vaginal vault, and transferring the tip into 0.4 ml complete DMEM. Viral titers were determined by a 96-well plate plaque assay on ICP0-complementing L7 cells cultured in complete DMEM containing 0.5% methlycellulose. After two to three days of incubation in each plaque assay, cell monolayers were stained with a solution of 20% methanol and 0.1% crystal violet and plaques were counted.

### Purification of baculovirus-expressed gD-2 and GFP

The methods that were employed to express and purify recombinant gD-2_306t_ and GFP proteins from baculovirus-infected insect cells are described, as follows.

#### i. Purification of gD-2306t antigen

The gD-2_306t_ protein engineered by Nicola, et al. (1996) possesses an N-terminal honeybee melittin secretion signal in place of gD-2's leader peptide, followed by amino acids 1–306 of the mature gD-2 protein and a C-terminal His_6_ affinity-purification tag [34]. The gD-2_306t_ protein was isolated from a flask containing 2×10^8^ High Five™ insect cells that had been inoculated 48 hours earlier with 2 pfu per cell of gD-2_306t_ -expressing baculovirus and incubated while shaking at 27°C. Baculovirus-infected cells were removed by centrifugation, and secreted gD-2_306t_ protein was purifed from supernatants by dialysis against an excess of 20 mM Tris pH 8.0, 300 mM NaCl, and 10% glycerol overnight. Imidazole was added to the dialyzed supernatant to a concentration of 10 mM prior to affinity purification on a HisTrap™ HP column (GE Healthcare Biosciences, Piscataway, NJ) using an ÄKTApurifier™ fast-performance liquid chromatography system (GE Healthcare Biosciences). The gD-2_306t_ protein was eluted from the column with 300 mM imidazole, and purity was verified at >90% by SDS-PAGE and Coomassie blue staining. Aliquots of gD-2_306t_ were stored at −80°C until use.

#### ii. Purification of GFP (irrelevant) antigen

A C-terminal, His-tagged GFP coding sequence was created by PCR amplification off of a pEGFP-C1 plasmid (Clontech Laboratories, Mountain View, CA), and was introduced into the pFastBacHtc vector (Invitrogen Corporation, Carlsbad, CA) via Nco I and Xho I restriction sites. A stock of His-GFP virus was constructed in Sf9 insect cells according to the manufacturer's protocol. Recombinant GFP protein was isolated from His-GFP-infected High Five™ insect cells per the same protocol used to isolate gD-2_306t_, except that His-GFP-infected insect cells were harvested by centrifugation 48 hours post-infection, lysed, and the lysate was applied to a HisTrap™ HP column (GE Healthcare). Fractions containing purified GFP (which were visibly lime-green) were combined, aliquoted, and stored at −80°C until use.

### Analysis of serum antibody responses to HSV-2 vaccines

Mice were bled on Day 50 or 60 post-inoculation by collecting blood from the right retroorbital sinus with heparinized, Natelson blood collecting tubes. The serum fraction was collected and stored at −80°C until use in 1 of 4 assays for estimating the abundance of HSV-2-specific antibody. Each method is described, as follows.

#### i. gD-2-antibody-capture ELISA

High-binding EIA 96-well plates (Costar, Corning, NY) were coated overnight at 4°C with 100 µl per well of sodium carbonate buffer (pH 9.6) containing 1.5 µg per ml gD-2_306t_ protein [34]. Wells were blocked for 2 hours with 400 µl of 2% dry milk dissolved in PBS+0.02% Tween-20 (polyoxyethylene-20-sorbitan monolaurate), hereafter referred to as PBS-T buffer. Mouse serum was diluted 1∶100 in PBS+1% fetal bovine serum+0.02% Tween-20. After discarding blocking buffer from ELISA plates, duplicate 100-µl samples of 1∶100 diluted mouse serum were added to gD_2306t_ -coated wells and were incubated for 2 hours. ELISA plates were rinsed seven times with an excess of PBS-T buffer prior to the addition of 100 µl secondary antibody diluted 1∶2500 in PBS-T buffer; the secondary antibody was alkaline phosphatase-conjugated rabbit anti-mouse γ chain (Rockland Immunochemicals, Gilbertsville, PA). After allowing 1 hour, secondary antibody was rinsed from plates seven times with PBS-T buffer, and 200 µ1 of p-nitrophenyl phosphate substrate (Sigma Chemical Co., St. Louis, MO) was added to each well, and colorimetric development (OD_405_) was measured after a 30 minute incubation at room temperature.

#### ii. Antiserum-dependent neutralization of HSV-2 virion infectivity

Two µl of each serum sample was added to a single well in the top row of a microtiter plate containing 91 µl of complete DMEM to achieve an initial 1∶46 dilution. Serial 0.33-log dilutions were achieved by serial transfer of 43 µl into 50 µl diluent (final volume = 93 µl) from the top to the bottom of the plate. A virus-complement mixture was created by diluting guinea pig complement (Rockland Immunochemicals, Gilbertsville, PA) 1∶50 in complete DMEM and adding HSV-2 MS to a concentration of 3,500 pfu per ml. The HSV-2 neutralization assay was initiated by combining 50 µl of the virus-complement mixture with each serum dilution (50 µl) and incubating at 37°C. After 2 hours, 100 µl of a suspension containing 4×10^6^ Vero cells per ml was added to each well, and microtiter plates were incubated for 48 hours to allow HSV-2 plaques to form. Cell monolayers were fixed and stained with a 20% methanol, 0.1% crystal violet solution. The HSV-2 neutralizing titer of each serum sample was considered to be the reciprocal of the largest serum dilution that reduced HSV-2's cytopathic effect in Vero cell monolayers by at least 50%.

#### iii. Mouse antiserum-staining of HSV-2 plaques

Vero cell monolayers were seeded in 12-well plates at a density of 5×10^6^ Vero cells and were inoculated 4 hours later with 40 pfu per well of HSV-2 MS. At 32 hours p.i., Vero cell monolayers containing well-spaced HSV-2 plaques were fixed for 20 minutes with a 2% formaldehyde-2% sucrose solution and were permeabilized for 10 minutes with 90% methanol. HSV-1 Fc-γ receptors (glycoprotein E-I heterodimers; [89]) and non-specific-binding sites were blocked with PBS containing 0.5% fetal bovine serum and 10 µg per ml each of human γ-globulin, donkey γ-globulin, and goat γ-globulin (PBS-F-Ig). Fixed monolayers containing HSV-2 plaques were incubated for 6 hours with a 1∶5,000 dilution of mouse antiserum, excess antibody was removed with two rinses, and cells were incubated for 2 hours in a 1∶1,000 dilution of Alexa Fluor 594-conjugated goat antibody specific for the Fc region of mouse IgG (Molecular Probes, Eugene, OR). Excess secondary antibody was removed by two rinses, and cells were photographed using a TE2000 inverted fluorescent microscope (Nikon Instruments, Lewisville, TX) and DP72 digital camera (Olympus America Inc., Center Valley, PA).

#### iv. Flow cytometric measurement of pan-HSV-2 IgG antibody levels

Five 100 mm dishes of HSV-2 infected cells were harvested 18 hours after inoculation with 2.5 pfu per cell. Cells were trypsinized, resuspended in PBS+0.5% FBS (PBS-F), centrifuged, and resuspended in 2% formaldehyde+2% sucrose for 20 minutes. Fixed cells were centrifuged, resuspended in 90% methanol for 10 minutes, centrifuged, and resuspended in PBS-F. Five dishes of uninfected Vero cells were identically processed in parallel. Suspensions of fixed and permeabilized cells were passed through 25 gauge needles to disperse cells into a uniform, single-cell suspension. Cells were brought to a concentration of 5.6×10^6^ cells per ml in PBS-F-Ig block solution, and 90 µl aliquots of uninfected or virus-infected cells were placed in a matched pair of tubes containing 10 µl of each 1∶500 dilution of pre-absorbed mouse antiserum (i.e., 1∶500 diluted serum was incubated overnight with 0.5×10^6^ uninfected Vero cells). After a 6-hour incubation with 1∶5,000-diluted mouse serum, cells were centrifuged and rinsed twice with PBS-F to remove excess mouse serum, and were then incubated with a 1∶2,000 dilution of allophycocyanin-conjugated goat antibody specific for the Fc region of mouse IgG (Jackson Immunoresearch Laboratories, Inc., West Grove, PA). After 1 hour, excess secondary antibody was removed by centrifugation and two rinses with PBS-F. Labeled cells were analyzed in an Accuri C6 flow cytometer (Accuri Cytometers, Inc., Ann Arbor, MI) using CFlow software (Accuri Cytometers Inc.). Background fluorescence was defined as the average mean fluorescent intensity (MFI) observed in uninfected cell suspensions incubated with naïve serum. The relative abundance of pan-HSV-2 IgG in serum was calculated as, (MFI _HSV-2 cells_−MFI _UI cells_)÷background (Fig. S4).

In the process of developing the flow cytometry-based assay, a panel of 12 representative mouse serum samples was tested on 4 independent occasions. Within each of these serum samples, the absolute estimate of pan-HSV-2 IgG abundance varied by an average 30% between independent trials. However, the rank-order of pan-HSV-2 IgG abundance was invariant among the 12 samples between independent trials.

### In vivo imaging of HSV-2 MS-GFP and HSV-2 MS-luciferase infections

#### i. HSV-2 MS-GFP challenge experiments

Fluorescent photographs of the eyes and faces of mice inoculated with 100,000 pfu per eye of HSV-2 MS-GFP was visualized using a TE2000 inverted fluorescent microscope (Nikon Instruments) fitted with a DP72 digital camera (Olympus America). Mice were anesthetized by i.p. administration of xylazine (6.6 mg/kg) and ketamine (100 mg/kg) and placed face on a clear petri dish. Photographs of the left side of mouse faces were obtained by capturing 20 to 30 photographs with a 2× objective that spanned the face, and merging individual images using the photomerge feature of Photoshop CS3 software (Adobe Systems Incorporated, San Jose, CA).

#### ii. HSV-2 MS-luciferase challenge experiments

Luciferase expression in mice inoculated with 100,000 pfu per eye or 500,000 pfu per vagina of HSV-2 MS-luciferase was visualized using an IVIS® Lumina II bioluminescent imager (Caliper Instruments, Hopkinton, MA). Mice were anesthetized by i.p. administration of xylazine (6.6 mg/kg) and ketamine (100 mg/kg), injected with 3 mg D-luciferin substrate (Gold BioTechnology, Inc., St. Louis, MO), positioned inside the instrument, and a 120-second exposure was captured. Longer and shorter exposures between 30 and 300 seconds were tested, but these had no significant effect on the relative differences between groups. Images were manipulated in Living Image v3.1 software (Caliper Instruments), and were scaled in a manner different than the default settings, specified as follows: ***1.*** binning (a signal : noise manipulation) was eliminated by setting to a value of ‘1;’ ***2.*** colored representations of light emissions were set to a logarithmic scale which allows visualization of the entire range of light emissions detected by the instrument; and ***3.*** the upper and lower limits of the scale were always set to the same boundaries (1 to 1000) such that the graphic representations of results were comparable between tests. Finally, quantitation of light emission from HSV-2 MS-luciferase-challenged animals was calculated within the Living Image v3.1 program (Figures 6B and 6D) by copying identically-sized “region of interest” boxes between Day 2, 4, and 6 measurements of all experiments at the conclusion of the study.

### Mathematical and statistical analysis of results

Viral titers were transformed by adding a value of 1 such that all data could be analyzed on a logarithmic scale. The significance of differences between multiple treatment groups was compared by one-way analysis of variance (ANOVA) followed by Tukey's post hoc t-test. The data that was graphed and statistically compared were the logarithms of ***i.*** HSV-2 shedding (e.g., pfu per vagina), ***ii.*** gD-specific IgG abundance, ***iii.*** neutralizing antibody titer, ***iv.*** pan-HSV-2 IgG levels. or ***v.*** luciferase activity (light emission) The correlation (goodness-of-fit) between pan-HSV-2 antibody levels and reductions in vaginal shedding following HSV-2 MS challenge was evaluated by regression analysis. The significance of differences in survival frequency between immunization groups was determined by Fisher's Exact Test. The significance of differences in 0ΔNLS- versus gD-2-immunized mice was compared by a two-sided, paired t-test for the following measurements: ***i.*** reductions in HSV-2 vaginal shedding (Fig. 4C); ***ii.*** pan-HSV-2 IgG levels (Fig. 5C); ***iii.*** reductions in luciferase activity (Fig. 6). The significance of differences in percent survival following HSV-2 MS challenge of 0ΔNLS-immunized mice and gD-2-immunized mice was compared by a two-sided Student's t-test. Statistical analyses were performed using Instat v3.0 software (Graphpad Software, La Jolla, CA) and Microsoft Excel. The quantitative relationship between color development in ELISA and abundance of gD-specific IgG antibody was defined by a hyperbolic tangent-based standard curve of the form x = x_50_+ΔX • arctan 
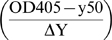
, as described elsewhere [52], [90].

## Supporting Information

Figure S1 **Shedding of HSV-2 MS and 0ΔNLS from the site of inoculation.** (**A**) HSV-2 shedding from the vaginas of mice on Days 2 and 4 p.i. with 500,000 pfu per vagina of wild-type HSV-2 MS or 0ΔNLS. (**B**) HSV-2 shedding from the eyes of mice on Days 2 and 4 p.i. with 100,000 pfu per eye of HSV-2 MS or 0ΔNLS. A single asterisk (*) denotes p<0.05 and a double asterisk (**) denotes p<0.001 that titers of HSV-2 0ΔNLS shed from the vagina or eyes were equivalent to titers shed at the same site on the same day by mice inoculated with HSV-2 MS.
(TIF)

Figure S2 **Mice immunized with HSV-2 0ΔNLS are resistant to HSV-2 ocular challenge.** On Day 56 p.i., HSV-2 0ΔNLS- and MS-immunized mice were challenged with 100,000 pfu per eye of HSV-2 MS. (**A**) HSV-2 shedding from the eyes between Days 1 and 3 post-challenge in naïve mice (n = 10) versus mice inoculated in the rear footpads with HSV-2 0ΔNLS (n = 5). (**B**) HSV-2 shedding from the eyes of naïve mice versus mice inoculated in the eyes, nose, or vagina with HSV-2 0ΔNLS (n = 5 per group). A single asterisk (*) denotes p<0.05 and a double asterisk (**) denotes p<0.001 that HSV-2 shedding was equivalent to naïve controls on that day. (**C**) Survival frequency of naïve mice (n = 10) versus immunized mice (n = 5 per group) one month after HSV-2 challenge of the eyes. A double asterisk (**) denotes p<0.001 that survival frequency was equivalent to naïve mice.
(TIF)

Figure S3 **Resistance of naïve versus immunized mice to ocular HSV-2 infection.** On Days 80, 90, or 100 p.i., mice were challenged with 100,000 pfu per eye of HSV-2 MS (n = 5 per group). The summated results from all three experiments are presented in each panel (∑n = 15 per group). (**A**) Ocular HSV-2 shedding between Days 1 and 7 post-challenge in naïve mice (medium-treated) versus mice immunized with gD-2_1-306t_ or HSV-2 0ΔNLS. (**B**) Ocular HSV-2 shedding in naïve mice versus mice immunized with GFP or HSV-2 MS. A single asterisk (*) denotes p<0.05 and a double asterisk (**) denotes p<0.001 that HSV-2 shedding was equivalent to naïve mice on that day. (**C**) Mean ± sem reduction in HSV-2 shedding on Days 1–5 post-challenge relative to the average titer of HSV-2 shed by naïve mice on that day (n = 605 per group). (**D**) Survival frequency over time following HSV-2 MS challenge of the eyes. A double asterisk (**) denotes p<0.001 that survival frequency was equivalent to naïve mice.
(TIF)

Figure S4 **Flow cytometry measurement of serum levels of pan-HSV-2 IgG.** (**A**) Summary of procedure. The immunofluorescent background of each serum dilution was defined as the average of the mean fluorescent intensity (MFI) of uninfected cell suspensions incubated with that dilution of naïve serum. (**B**) Flow cytometric analysis of 5-fold dilution series of antiserum samples (n = 3 samples per dilution).
(TIF)

Figure S5 **Description of HSV-2 MS-GFP and HSV-2 MS-luciferase.** (**A**) Schematic of CMV-GFP and CMV-luciferase expression cassettes introduced into the non-essential *LAT* gene of HSV-2 MS-GFP and MS-luciferase, respectively. These gene expression cassettes replaced bases 119,359–119,530 of the *LAT* promoter. (**B**) Southern blot analysis of NotI-digested plasmid DNA (shown on left) or NotI-digested viral DNA (shown on right). The plasmid pUC-HSV-2-LAT contains the wild-type *LAT* gene. The plasmids pUC-ΔLAT-GFP and pUC-ΔLAT-luciferase were the plasmid precursors of HSV-2 MS-GFP and HSV-2 MS-luciferase, respectively. NotI-digested cellular DNA was derived from Vero cells that were uninfected (UI) or were harvested 18 hours after inoculation with 2.5 pfu per cell of HSV-2 MS, MS-GFP, or MS-luciferase. A *LAT* promoter-specific oligonucleotide (5′-ccctgtgtcattgtttacgtggccgcgggccagcagacgg-3′) was hybridized to Southern blots, which hybridized upstream of the PvuII – BspEI deletion in the *LAT* gene, and which verified that the gene expression cassettes in pUC-ΔLAT-GFP and pUC-ΔLAT-luciferase were transferred into the intended locus in the HSV-2 genome.
(TIF)

Figure S6 **Spread of HSV-2 MS-GFP infection between Days 1 and 7 after challenge of naïve versus HSV-2 0ΔNLS-immunized mice.** Progression of the spread of GFP expression across the faces of naïve and 0ΔNLS-immunized mice challenged with 100,000 pfu per eye of HSV-2 MS-GFP, as visualized on Days 1, 3, 5, and 7 post-challenge. These experiments were performed on n = 3 mice per group, and the progression of infection is shown in a single representative mouse per group.
(TIF)

Table S1 (DOC)
