# Supplementary figures and images for: A Live-Attenuated HSV-2 ICP0 − Virus Elicits 10 to 100 Times Greater Protection against Genital Herpes than a Glycoprotein D Subunit Vaccine
Source: PLoS One. 2011 Mar 11;6(3):e17748. doi: 10.1371/journal.pone.0017748 (PMC3055896; doi:10.1371/journal.pone.0017748)

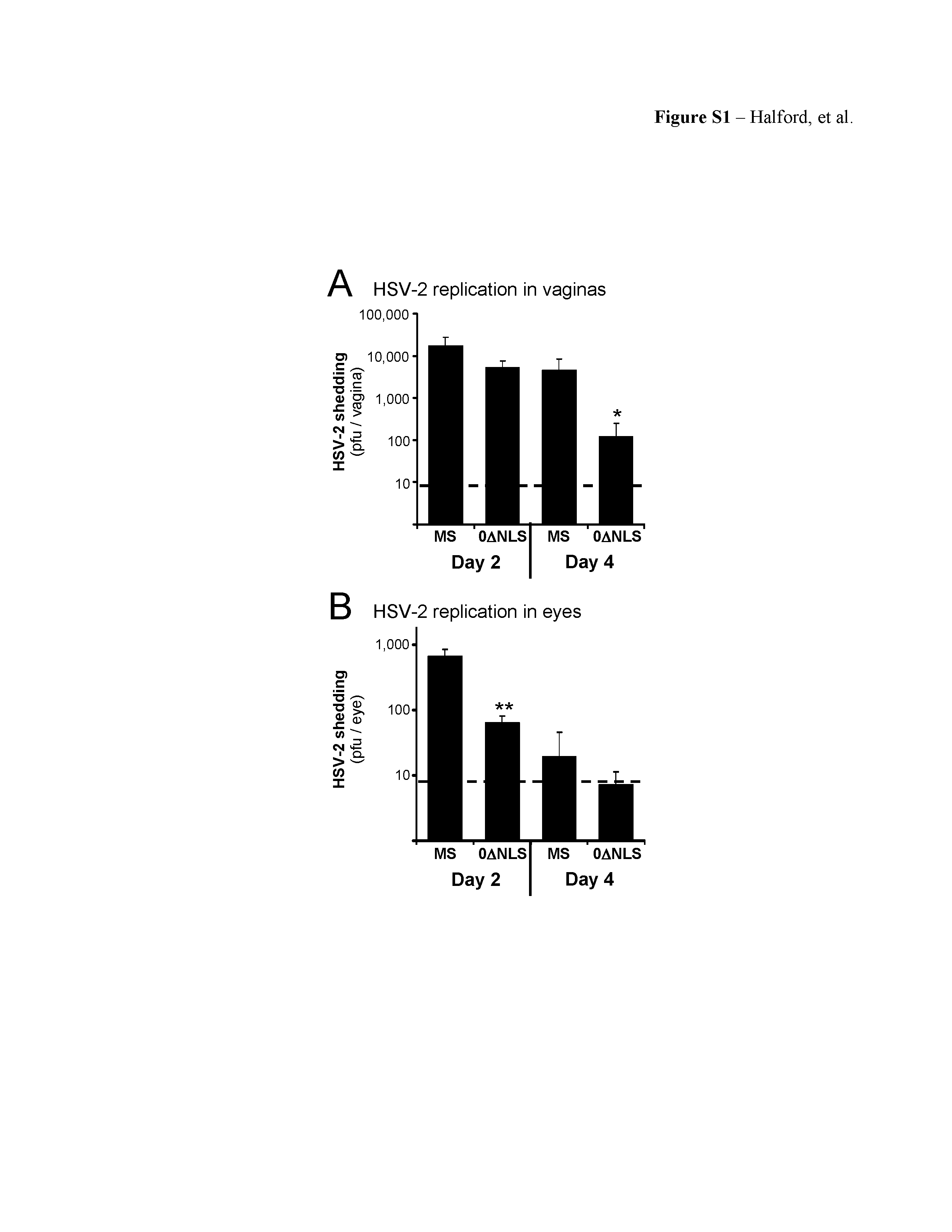

Supplement: Figure S1 — Shedding of HSV-2 MS and 0ΔNLS from the site of inoculation. (A) HSV-2 shedding from the vaginas of mice on Days 2 and 4 p.i. with 500,000 pfu per vagina of wild-type HSV-2 MS or 0ΔNLS. (B) HSV-2 shedding from the eyes of mice on Days 2 and 4 p.i. with 100,000 pfu per eye of HSV-2 MS or 0ΔNLS. A single asterisk (*) denotes p<0.05 and a double asterisk (**) denotes p<0.001 that titers of HSV-2 0ΔNLS shed from the vagina or eyes were equivalent to titers shed at the same site on the same day by mice inoculated with HSV-2 MS. (TIF) [file pone.0017748.s001.tif]

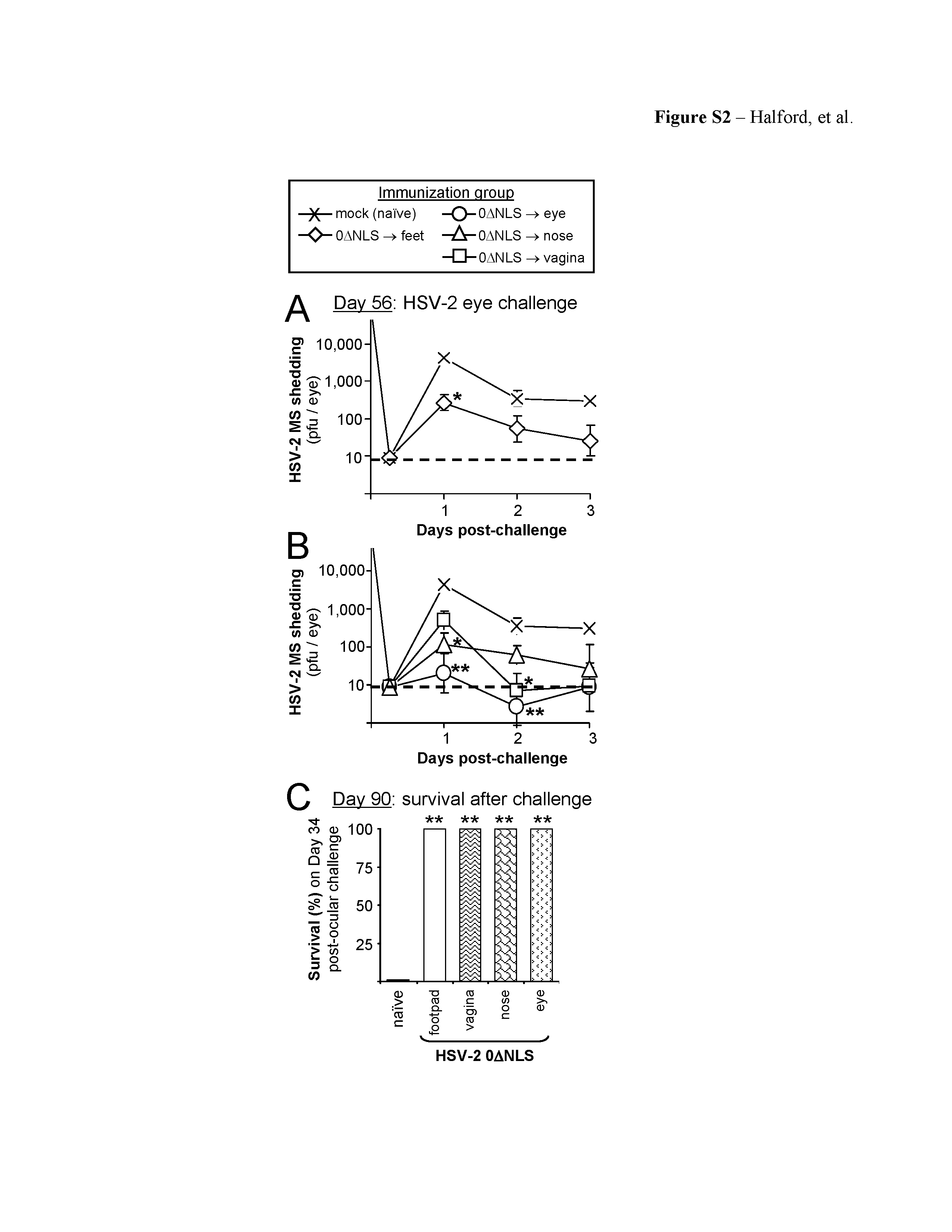

Supplement: Figure S2 — Mice immunized with HSV-2 0ΔNLS are resistant to HSV-2 ocular challenge. On Day 56 p.i., HSV-2 0ΔNLS- and MS-immunized mice were challenged with 100,000 pfu per eye of HSV-2 MS. (A) HSV-2 shedding from the eyes between Days 1 and 3 post-challenge in naïve mice (n = 10) versus mice inoculated in the rear footpads with HSV-2 0ΔNLS (n = 5). (B) HSV-2 shedding from the eyes of naïve mice versus mice inoculated in the eyes, nose, or vagina with HSV-2 0ΔNLS (n = 5 per group). A single asterisk (*) denotes p<0.05 and a double asterisk (**) denotes p<0.001 that HSV-2 shedding was equivalent to naïve controls on that day. (C) Survival frequency of naïve mice (n = 10) versus immunized mice (n = 5 per group) one month after HSV-2 challenge of the eyes. A double asterisk (**) denotes p<0.001 that survival frequency was equivalent to naïve mice. (TIF) [file pone.0017748.s002.tif]

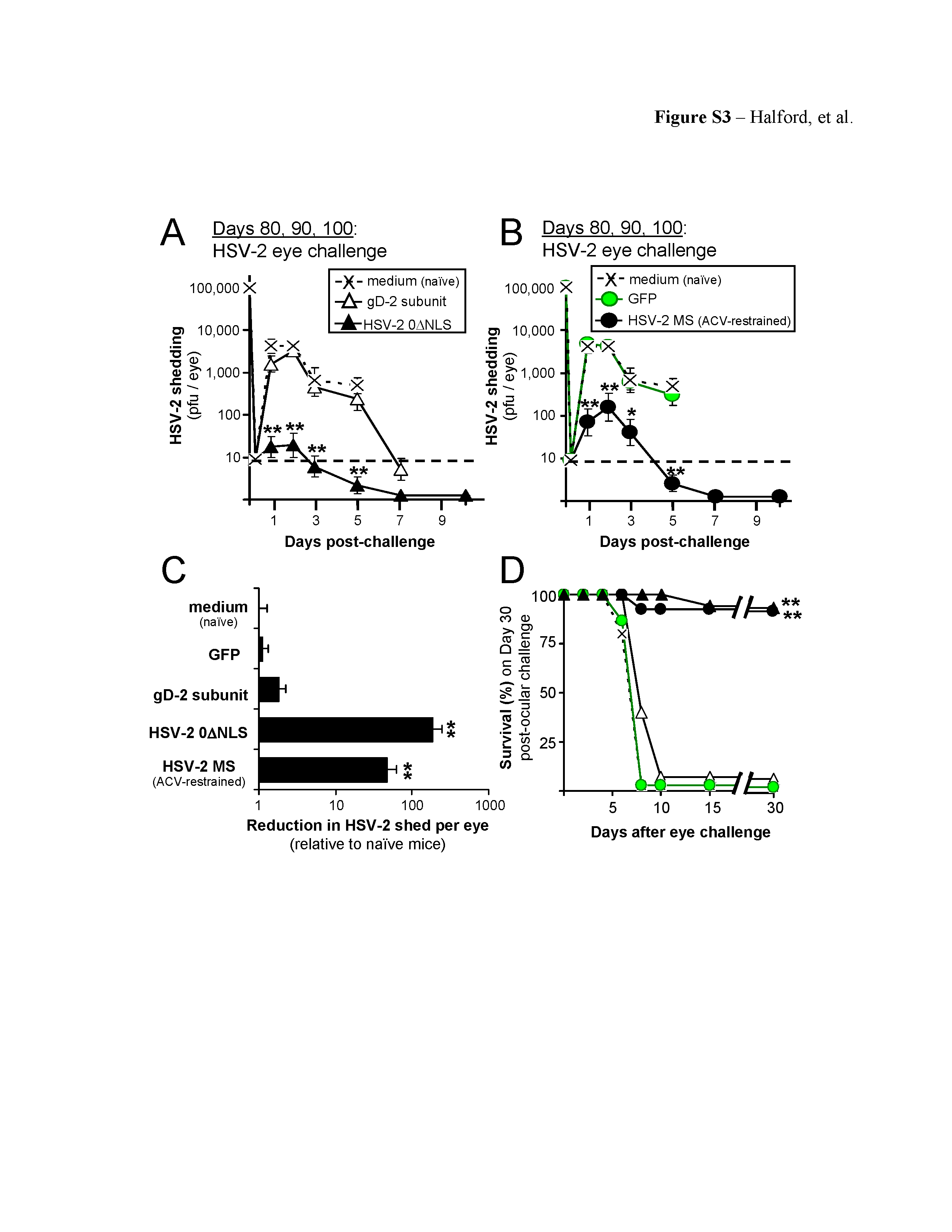

Supplement: Figure S3 — Resistance of naïve versus immunized mice to ocular HSV-2 infection. On Days 80, 90, or 100 p.i., mice were challenged with 100,000 pfu per eye of HSV-2 MS (n = 5 per group). The summated results from all three experiments are presented in each panel (∑n = 15 per group). (A) Ocular HSV-2 shedding between Days 1 and 7 post-challenge in naïve mice (medium-treated) versus mice immunized with gD-21-306t or HSV-2 0ΔNLS. (B) Ocular HSV-2 shedding in naïve mice versus mice immunized with GFP or HSV-2 MS. A single asterisk (*) denotes p<0.05 and a double asterisk (**) denotes p<0.001 that HSV-2 shedding was equivalent to naïve mice on that day. (C) Mean ± sem reduction in HSV-2 shedding on Days 1–5 post-challenge relative to the average titer of HSV-2 shed by naïve mice on that day (n = 605 per group). (D) Survival frequency over time following HSV-2 MS challenge of the eyes. A double asterisk (**) denotes p<0.001 that survival frequency was equivalent to naïve mice. (TIF) [file pone.0017748.s003.tif]

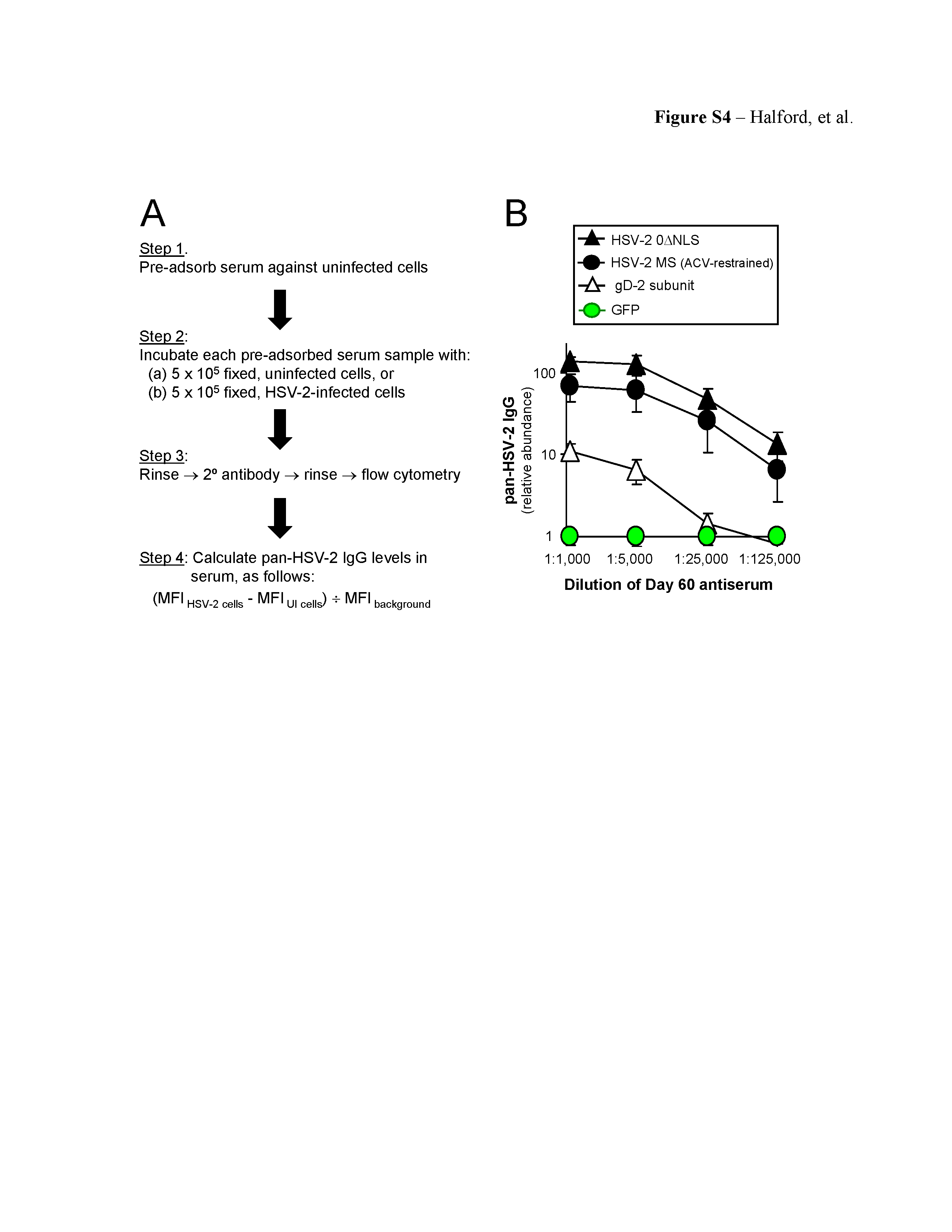

Supplement: Figure S4 — Flow cytometry measurement of serum levels of pan-HSV-2 IgG. (A) Summary of procedure. The immunofluorescent background of each serum dilution was defined as the average of the mean fluorescent intensity (MFI) of uninfected cell suspensions incubated with that dilution of naïve serum. (B) Flow cytometric analysis of 5-fold dilution series of antiserum samples (n = 3 samples per dilution). (TIF) [file pone.0017748.s004.tif]

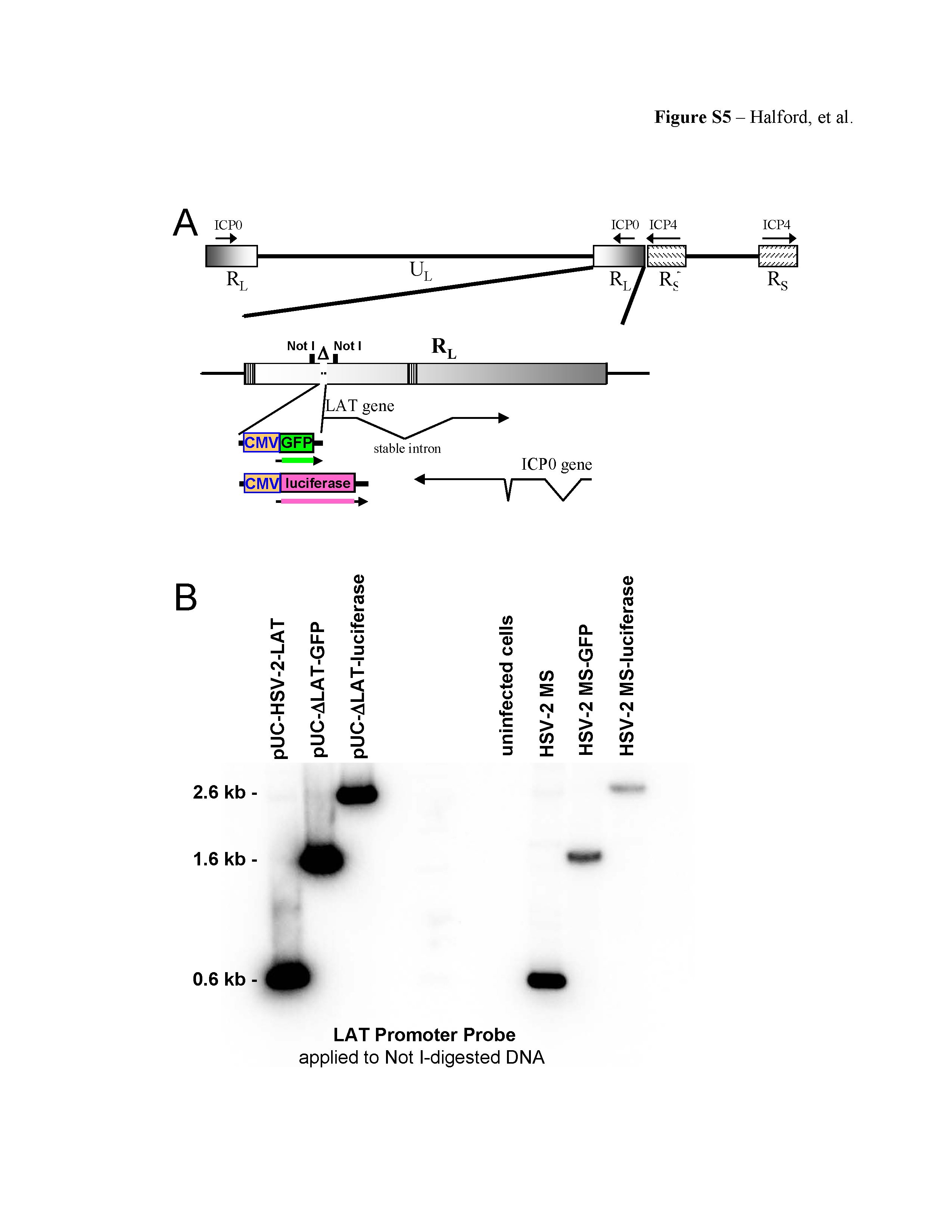

Supplement: Figure S5 — Description of HSV-2 MS-GFP and HSV-2 MS-luciferase. (A) Schematic of CMV-GFP and CMV-luciferase expression cassettes introduced into the non-essential LAT gene of HSV-2 MS-GFP and MS-luciferase, respectively. These gene expression cassettes replaced bases 119,359–119,530 of the LAT promoter. (B) Southern blot analysis of NotI-digested plasmid DNA (shown on left) or NotI-digested viral DNA (shown on right). The plasmid pUC-HSV-2-LAT contains the wild-type LAT gene. The plasmids pUC-ΔLAT-GFP and pUC-ΔLAT-luciferase were the plasmid precursors of HSV-2 MS-GFP and HSV-2 MS-luciferase, respectively. NotI-digested cellular DNA was derived from Vero cells that were uninfected (UI) or were harvested 18 hours after inoculation with 2.5 pfu per cell of HSV-2 MS, MS-GFP, or MS-luciferase. A LAT promoter-specific oligonucleotide (5′-ccctgtgtcattgtttacgtggccgcgggccagcagacgg-3′) was hybridized to Southern blots, which hybridized upstream of the PvuII – BspEI deletion in the LAT gene, and which verified that the gene expression cassettes in pUC-ΔLAT-GFP and pUC-ΔLAT-luciferase were transferred into the intended locus in the HSV-2 genome. (TIF) [file pone.0017748.s005.tif]

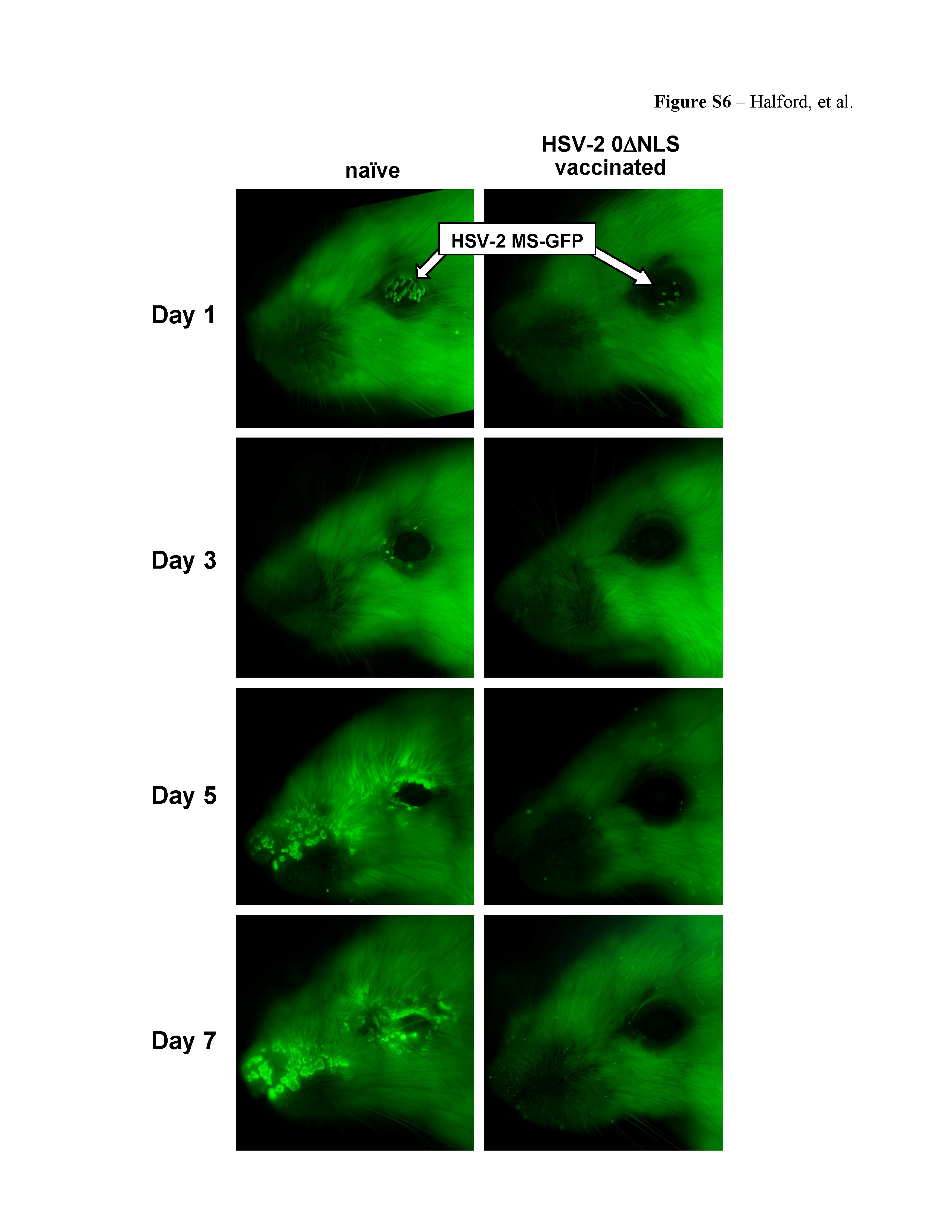

Supplement: Figure S6 — Spread of HSV-2 MS-GFP infection between Days 1 and 7 after challenge of naïve versus HSV-2 0ΔNLS-immunized mice. Progression of the spread of GFP expression across the faces of naïve and 0ΔNLS-immunized mice challenged with 100,000 pfu per eye of HSV-2 MS-GFP, as visualized on Days 1, 3, 5, and 7 post-challenge. These experiments were performed on n = 3 mice per group, and the progression of infection is shown in a single representative mouse per group. (TIF) [file pone.0017748.s006.tif]
