# Supplementary material for: A Live-Attenuated HSV-2 ICP0 − Virus Elicits 10 to 100 Times Greater Protection against Genital Herpes than a Glycoprotein D Subunit Vaccine
Source: PLoS One. 2011 Mar 11;6(3):e17748. doi: 10.1371/journal.pone.0017748 (PMC3055896; doi:10.1371/journal.pone.0017748)
Supplement: Table S1 — (DOC) [file pone.0017748.s007.doc]

**Table S1. Survival rates in individual HSV-2 MS challenge experiments.**

| **HSV-2 MS ocular challenge** (100,000 pfu per eye) | | | | | | | |
| --- | --- | --- | --- | --- | --- | --- | --- |
| **Date of Immunization** | **Exp** | **Days after immunization** | **naïve**^a^ | **GFP** | **gD-2** | **0ΔNLS** | **MS** |
| 6-29-08 | 1 | 45 ^b^ | 0 / 5 ^c^ | - | - | - | 5 / 5 |
| 7-28-08 | 2 | 60 | 0 / 5 | - | - | - | 6 / 6 |
| 9-23-08 | 3 | 70 | 0 / 10 | - | 0 / 10 | - | - |
| 11-18-08 | 4 | 50 | 0 / 5 | - | 0 / 5 | - | 4 / 5 |
| 5-18-09 | 5 | 45 | 0 / 4 | - | - | 4 / 4 | - |
| 5-28-09 | 6 | 30 | 0 / 5 | - | - | 5 / 5 | - |
| 8-5-09 | 7 | 50 | 0 / 8 | - | - | 12 / 12 | - |
| 8-17-09 | 8 | 70 | 0 / 7 | - | - | 8 / 8 | - |
| 9-14-09 | 9 | 80 | 0 / 15 | - | - | 9 / 9 | 12 / 13 |
| 10-21-09 | 10.A | 56 | 0 / 10 | - | - | 20 / 20 | 5 / 5 |
| 11-13-09 | 11.A | 80 | 0 / 5 | 0 / 5 | 0 / 5 | 4 / 5 | 4 / 5 |
| 11-13-09 | 12.A | 90 | 0 / 5 | 0 / 5 | 0 / 5 | 5 / 5 | 5 / 5 |
| 11-13-09 | 13.A | 100 | 0 / 5 | 0 / 5 | 0 / 5 | 5 / 5 | 5 / 5 |
| 11-9-09 | 14 | 140 | 0 / 5 | - | - | 4 / 4 | - |
| 9-22-09 | 15 | 190 | 0 / 5 | - | - | 3 / 3 | - |
| **Summated results:** | | | **0 / 99** | **0 / 15** | **0 / 30** | **79 / 80** **,**^‡^** | **46 / 49** ** |
|  |  |  |  |  |  |  |  |
| **HSV-2 MS vaginal challenge** (500,000 pfu per vagina) | | | | | | | |
| **Date of Immunization** | **Exp** | **Days after immunization** | **naïve** | **GFP** | **gD-2** | **0ΔNLS** | **MS** |
| 10-21-09 | 10.B | 56 | 0 / 10 | - | - | 20 / 20 | - |
| 11-13-09 | 11.B | 80 | 0 / 5 | 0 / 5 | 2 / 5 | 5 / 5 | 5 / 5 |
| 11-13-09 | 12.B | 90 | 0 / 5 | 0 / 5 | 0 / 5 | 5 / 5 | 5 / 5 |
| 11-13-09 | 13.B | 100 | 0 / 5 | 0 / 5 | 1 / 5 | 5 / 5 | 5 / 5 |
| **Summated results:** | | | **0 / 25** | **0 / 15** | **3 / 15 *** | **35 / 35** **,**^‡^** | **15 / 15** ** |
|  | | |  |  |  |  |  |
| **Overall survival frequency** | | | **naïve** | **GFP** | **gD-2** | **0ΔNLS** | **MS** |
|  | | | **0 ± 0% ^d^**  (n=19) | **0 ± 0%**  (n=6) | **8 ± 6%**  (n=8) | **99 ± 1%** ^¥^  (n=15) | **96 ± 3 %**  (n=11) |

^a^ Immunization status of mice at the time of HSV-2 MS challenge, which were vaccinated 45 to 190 days earlier with culture medium (naïve), GFP, gD-2, 0ΔNLS, or HSV-2 MS.

^b^ Number of days that elapsed between first exposure to the indicated immunogen and HSV-2 MS challenge of the eyes or vagina.

^c^ Frequency of mice that survived until 30 days after challenge with wild-type HSV-2 MS.

^d^ Mean ± sem of percent survival following HSV-2 MS challenge of the eyes or vaginas, as tabulated from the above column of survival frequencies. The number of independent experiments, n, on which this calculation is based is shown in parentheses.

* p < 0.05 that the survival frequency was equivalent to naïve mice following HSV-2 MS challenge, as determined by Fisher’s Exact Test.

** p < 10^-6^ that the survival frequency was equivalent to naïve mice following HSV-2 MS challenge, as determined by Fisher’s Exact Test.

**^‡^** p < 10^-8^ that survival frequency was equivalent between mice immunized with HSV-2 0ΔNLS versus a gD-2 subunit vaccine following HSV-2 MS challenge, as determined by Fisher’s Exact Test.

^¥^ p < 10^-15^ that percent survival was equivalent between mice immunized with HSV-2 0ΔNLS versus a gD-2 subunit vaccine following HSV-2 MS challenge, as determined by a two-sided Student's t-test.
